# Inhibition of Drp1 orchestrates the responsiveness of breast cancer cells to paclitaxel but insignificantly relieves paclitaxel-related ovarian damage in mice

**DOI:** 10.1038/s41598-023-49578-0

**Published:** 2023-12-20

**Authors:** Adel I. Alalawy, Mohamed Sakran, Fahad M. Alzuaibr, Maeidh A. Alotaibi, Mohamed E. El-Hefnawy, Abdulelah Y. Hazazi, Saad M. El-Gendy, Esraa A. Aidy, Heba Effat, Doha F. Ismail, Mohamed Hessien

**Affiliations:** 1 Department of Biochemistry, Faculty of Science, University of Tabuk, 71491 Tabuk, Saudi Arabia; 2 Division of Biochemistry, Faculty of Science, Tanta University, Tanta City, 31512 Egypt; 3 Biology Department, Faculty of Science, Tabuk University, Tabuk, Saudi Arabia; 4 King Faisal Medical Complex Laboratory, Ministry of Health, Taif, Saudi Arabia; 5 Department of Chemistry, Rabigh College of Sciences and Arts, King Abdulaziz University, Jeddah, Saudi Arabia; 6 Department of Cancer Biology, National Cancer Institute, Cairo University, Giza, Egypt; 7 Molecular Cell Biology Unit, Division of Biochemistry, Department of Chemistry, Faculty of Science, Tanta University, Tanta, 31512 Egypt

**Keywords:** Biochemistry, Cell biology, Chemical biology, Drug discovery, Molecular biology, Medical research, Risk factors

## Abstract

Chemoresistance and chemotherapy-related ovarian damage are well-reported in breast cancer (BC) young patients. Herein, the inhibition of the mitochondrial fission was invested to explore its chemosensitizing role in Paclitaxel (PTX)-resistant cells, and its ability to restore the ovarian integrity in mice receiving PTX or cisplatin chemotherapy. To establish these aims, PTX-resistance was generated in BC cells, which were treated with PTX in combination with Drp1 deficiency, via mdivi-1, or Drp1-specific siRNA transfection. Furthermore, the alterations in the ovarian structure and the endocrine-related hormones were explored in mice receiving repetitive doses of PTX or cisplatin. We found that combining PTX with mdivi-1 improved cell responsiveness to PTX, induced apoptosis- and autophagy-mediated cell death, and relieved cellular oxidative stress. Additionally, the expression of PCNA1 and cyclin B1 genes were downregulated, meanwhile, p53, p21, and mitochondrial fusion proteins (Mfu1&Mfu2) were increased. The in vivo investigations in mice demonstrated that PTX induced gonadotoxic damage similar to cisplatin, whereas dual treatment of mice with PTX+ mdivi-1 failed to restore their normal follicular count and the circulating levels of E2 and AMH hormones. These results suggested that combining Drp1 inhibition with PTX resensitized breast cancer cells to PTX but failed to offer enough protection against chemotherapy-related gonadotoxicity.

## Introduction

Mitochondria play a crucial role in the adaptability of cancer cells to both survival and hyperproliferation. Normally, mitochondrial dynamics, including mitochondrial fission and fusion, are desperately required to maintain their functions and shape. Both fusion and fission are regulated by a group of highly conserved proteins^1^, including the transmembrane mitofusins 1&2 (Mfn1/2), Opa1, and Dynamin-related protein (Drp1)^2,3^. The latter (Drp1) is a member of the large dynamins family, with an intrinsic guanosine triphosphatases (GTPase) activity, involved in dynamin self-assembly as a ring-like structure around mitochondrion, leading to their fragmentation^4–6^. Changes in the expression of Drp1 shift the equilibrium of mitochondrial biogenesis either towards fusion or fission. Also, Drp1 alterations are associated with pathological conditions including cancer development^7^. The availability of the quinazolinone derivative (mdivi-1), as a selective inhibitor of Drp1, and Drp1-specific short interfering RNA (siRNA) offers the opportunity to explore the role of Drp1 in different aspects of cancer, including drug resistance and progression. Although mdivi-1 is not currently in clinical trials, many reports have suggested its therapeutic potential, where it reversed some pathological conditions including cardiac hypertrophy in mice^8^, reduced angiotensin-II-induced hypertension^9^, stroke prevention^10^, and reversed neurodegenerative diseases^11^. In breast cancer cells, mdivi-1 induces spindle abnormalities and augments PTX cytotoxicity^12^. Also, mdivi-1 demonstrated a synergistic apoptotic effect with cisplatin in cisplatin-resistant ovarian, lung, and breast cancer cell lines^13,14^. Mechanistically, Drp1 inhibition mediates Bax/Bak-dependent mitochondrial outer membrane permeabilization, enhances mitochondrial fusion and alters oxygen consumption^15^. Furthermore, mdivi-1 (at 50 μM concentration), reversibly inhibited mitochondrial complex I-dependent O_2_ consumption and reversed electron transfer-mediated reactive oxygen species (ROS) production. These cumulative observations support the notion of targeting mitochondrial fission as a potential anticancer strategy. In this regard, triple-negative breast cancer accounts 15%, where its bad prognosis and treatment failure is attributed to PTX-resistance. Moreover, the chemotherapeutic drugs received by female cancer patients are the leading cause of gonadotoxicity and premature ovarian failure (POF) particularly in young women^16^. Although Drp1 inhibition was recently suggested as a cotherapeutic factor in PTX-resistant BC cells^12^, some of the mitochondrial related factors were inadequately investigated. Also, the potential of Drp1 inhibitor, mdivi-1, in relieving the chemotherapy-associated PTX-mediated gonadotoxicity is not addressed yet. Accordingly, this study was designed to explore how far Drp1 deficiency will improve the anticancer effect of PTX in PTX-resistant triple-negative breast cancer cells, in vitro, and whether the ovarian integrity will be restored in mice receiving repetitive doses of PTX.

## Materials and methods

### Key chemicals, cells, and treatments

Mdivi-1 was obtained from APExBIO Technology LLC, TX, USA, Unitaxel (Paclitaxel) was from Hikma, Egypt (Concentration 300 mg paclitaxel/50 ml), and cisplatin was purchased from Mylan, Viatris, PA, USA. Drp1-specific siRNA was obtained from Santa Cruz Biotech Inc, TX, USA, for siRNA transfection, Lipofectamine 2000 in OptiMem reduced serum media was from ThermoFisher Scientific, USA, and Wortmannin (Wort) was purchased from Toronto Research Chemicals (Canada). Monoclonal antibodies including Drp1, Mitofusin-1, and Mitofusin-2 were from Cell Signaling Technology, USA. MTT was from Sigma and cell culture media were from Lunza, Pharma Biotech. Breast cancer cells (MDA MB-231) were kindly provided by the Department of Cancer Biology, NCI, Cairo, Egypt.

### Cell culture and development of PTX-resistant cells

All methods were performed in accordance with the relevant guidelines and regulation. Breast cancer cells (MDA MB-231) were maintained in DMEM medium with L-glutamine, supplemented with 10% fetal bovine serum (FBS), and 1% penicillin/streptomycin in a humidified atmosphere, 5% CO_2_, and 95% air at 37 °C. In some experiments, cells were either grown in Earle’s Balanced Salt Solution (EBSS) media, for 4 h or treated with Wort, for 24 h, to induce or inhibit autophagy flux, respectively. To establish PTX-resistant phenotype, cells were grown in 10^−3^ of the PTX IC_50_ concentration for 2 weeks. During this period, dead (floating) cells were discarded upon replacing the old media with fresh media containing the low PTX concentration. Upon reaching subconfluent (after ≈5–6 days), cells were harvested by trypsin/EDTA and reseeded into new flasks until the end of the 2 weeks. PTX resistance was authenticated by measuring cell’s IC_50_, relative to the corresponding value of regular cells.

### Cell transfection with Drp1-specific siRNA

For Drp1 silencing, cells were grown at a density 2 × 10^5^ cells/well in 1.8 ml antibiotic/antimycotic free DMEM media, supplemented with 10% FBS. After incubation, until 60% confluency, cells were transfected with 40 pM siRNA oligonucleotides duplex, targeting Drp1 (Table 1), and the control cells, diluted in transfection media, mixed with transfection reagents and cells were incubated again for at least 6 h. The transfection protocol was carried out along with non-specific siRNA (siRNA-A) as a negative control^17^.

**Table 1:** Gene names, and oligonucleotide primers and Drp1-specific siRNA sequences used for quantitative expression analysis, and cell transfection.

Genes	Primer sequence
MDR1	F. 5′.CCAAAGCCAAAATATCAGC-3′ R. 5′-TTCCAATGTGTT CGGCAT-3′
Drp1	F. 5′. GATGCCATAGTTGAAGTGGTGAC-3′ R. 5′CCACAAGCATCAGCAAAGTCTGG-3′
Drp1 siRNA	F.5′. CCGUGACAAAUGAAAUGGUACAUAA.3′ R. 5. UUAUGUACCAUUUCAUUUGUCACGG. 3′
Cyclin D N1A (p21)	F. 5′. GAG CAG TGCCC GAG TTAAGG. 3′ R. 5′. TGGAACAGGTCGGACATCAC.3′
p53	F. 5′. CCCCTCCATCCTTTCTTCT. 3′ R. 5′. CATGAGCCAGATCAGGGACTG.3′
Cyclin B1	F. 5′. AAAGGCGTAACTCGAATGGA3′ R. 5′: -CCGACCTTTTATTGAAGAGCA-3′
PCNA1	F. 5′. ATATTAGCTCCAGCGGTGTAAA 3′ R. 5′ ACATCTGCAGACATACTGAGTG 3′
β*-Actin*	F.5′. ATCTGGCACCACACCTTCTA‐3′ R.5′. CGTCATACTCCTGCTTGCTG‐3′

*F* forward, *R* reverse.

### Cell metabolic activity assay

Drugs (PTX or mdivi-1) cytotoxicity and IC_50_ were determined using (3-(4,5-dimethylthiazol-2-yl)-2,5-diphenyltetrazoliumbromide thiazolyl (MTT) assay^18^. Briefly, cells were cultured at 2 × 10^4^ cells/well in 96-well plates. After overnight incubation at 5% CO_2_ and 37 °C, the media was replaced with fresh media containing different concentrations of PTX or mdivi-1, and the plates were incubated at 37 °C and 5% CO_2_ for 24 h. Cells were then labeled with 20 μl of MTT solution (5 mg/ml in PBS) per well, followed by 5 min shaking, after which they were incubated in the dark for 4 h. The medium was then removed, Isopropanol was added, and the absorbance of wells was measured at 570 nm.

### Apoptosis assay and autophagy assessment

Annexin V-FITC kit (Miltenyi Biotec, Auburn, CA, USA) was utilized to assess apoptosis as previously described^19^, following the manufacturer’s guidelines. Briefly, subconfluent cells were recovered by trypsinization and centrifuged at 1000 rpm for 5 min. The cell pellet was resuspended in 1 ml PBS and incubated with 0.25 μg/ml Annexin V in 1× binding buffer for 15 min, followed by two washes with Wash Buffer. Cells were resuspended again in a binding buffer containing 0.5 μg/ml of Propidium Iodide (PI) and then subjected to flow cytometry (BC, Novus). The data were analyzed by Kaluza software. To investigate the autophagy effects of drugs, the level of LC3II protein was determined using anti-LC3II antibody and flow cytometry. In parallel autophagy was induced by incubating PTX-resistant cells in EBSS for 4 h or inhibited by treating cells with 100 nM Wort as positive and negative controls, respectively.

### Cell cycle analysis

After cell treatments, adherent cells were collected, washed with PBS, and fixed with 70% ethanol (in PBS v/v). After incubation at 4 °C for at least 2 h, cells were washed with PBS and stained with PBS containing PI (50 μg/ml, Triton X-100, and RNase A) for 30 min at room temperature in a dark place. The cell suspension was filtered and then analyzed by Accuri C6 flow cytometer (Becton Dickinson, Sunnyvale, CA, USA) to determine cell populations in different cell cycle phases.

### ELISA measurements for redox functions and hormones

The colorimetric Hydrogen peroxide assay ELISA kit (Abcam, USA) was used to assess the level of H2O2 in cell lysates. Cells were collected, washed, lysed, and centrifuged at 4 °C at 12,000× *g* for 10 min. The lysate was utilized to determine H2O2 following the manufacturer’s protocol. Similarly, the activity of superoxide dismutase (SOD) was determined using Superoxide Dismutase Assay Kit (Cayman, chemicals, USA), following the manufacturer’s instructions. Both estradiol (E2) and antimullerian hormone (AMH) were measured using Cobas (Roche Diagnostics GmbH, Mannheim, Germany), following the manufacturer’s guidelines.

### In vitro cell migration assay

In this assay, cells were grown in a 6-well plate up to confluency, where a free area “wound” was created in the cells monolayer and the plate was incubated in serum-free media. The scratch was imaged at 0 and 24 h to determine drug-treated cell migration rate compared to the untreated cells, where area changes were determined by using Image J.

### RNA isolation, cDNA synthesis, and gene expression analysis

Total RNA was extracted using a total RNA isolation Kit (GeneDireX, Inc), and the quantity and the integrity of RNAs were determined by measuring the absorbance ratio A260/A280. Next, RNA was reverse-transcribed using SuperScript II, and the obtained cDNA was employed as a template in relative quantitative PCR (RT-qPCR) to determine the expression of multidrug resistance gene (MDR1), cell cycle (p21, p53, cyclin B1 & PCNA1), and Drp1 genes. The sequences of gene-specific primer pairs are shown in Table 1. Amplification reactions were carried out in a 25 µl mixture containing 150 ng of forward and reverse primers and ROX High Reference Dye 1in a thermal cycler. The comparative CT method was used to quantify the expression of genes and this was normalized to the relative expression of β-actin. Changes in gene expression were expressed using the 2-DDCT method and the fold-changes, in triplicate experiments, were presented as mean (± SD).

### Immunoblotting of mitochondrial proteins

Standard immunoblotting protocol was employed to determine the expression of Drp1, Mfu1 and Mfu2. Cell lysate (20 μg) was mixed with sample buffer (0.125M Tris/HCl pH 6.8, 10% glycerol, 4% SDS, 0.25M DTT) and heated for 5 min at 95 °C. Next, proteins were resolved onto 12% polyacrylamide gel using protein electrophoresis and blotted onto nitrocellulose membranes. The membrane was blocked with 5% BSA for 30 min, hybridized with Drp1-, Mfu1-, or Mfu2-specific antibodies at 4 °C, washed with TBS containing 0.1% Tween-20, and then incubated for 2 h with the corresponding horseradish peroxidase-conjugated secondary antibody. Band intensities were quantitated using Image J.

### Transmission electron microscopy

For electron microscope imaging, cells were fixed in 2.5% glutaraldehyde in 0.1M phosphate buffer for 2 h at 4 ℃, followed by washing with 0.1M PBS (pH 7.4). Cells were then fixed with 1% osmium tetroxide and 1% potassium hexacyanoferrate (III) for 1 h and dehydrated by passing the specimen through increasing concentrations of ethanol (30–90%). Ultrasections (about 40 nm) of cells were embedded in Epon 812 resin (Fluka, Germany) and stained with lead citrate and uranyl acetate. Finally, sections were examined by TEM (JEOL, JEM-1400), and photographed at using the CCD camera (Model AMT).

### Computational analysis

Protein-small molecule docking recognition was utilized to access the interaction between PTX or mdivi-1 using SwissDock^20^. Mitochondrial ATP synthase, SOD, and Thioredoxin reductase type sequences were utilized to generate their PDB file by homology modeling using SWISS-MODEL (https://swissmodel.expasy.org). Accordingly, the molecular docking analysis was performed by molecular operating environment (MOE) software.

### Animal experiments

The in vivo experiment involved healthy adult female C57BL/6J mice (aged 8–10 weeks) obtained from The National Cancer Institute, Cairo University. Methods involving animal work were performed in accordance with ARRIVE guidelines (https://arriveguidelines.org) and the protocol was approved by the Institutional Animal Care and Use Committee of the Faculty of Science, Tanta University (ECL: 2022/A-87). Initially, the development of POF was authenticated in small investigative group of mice (n = 4), in which animals received 2 or 4 mg/kg cisplatin every other day for 15 days (8 doses). At the end of treatment period mice were sacrificed, ovaries were recovered to assess the ovarian damage and the complete development of POF. Histological POF signs were indicated by a decrease in the follicle number, the predominance of unhealthy follicles, and the existence of apoptosis in the granulosa cells. The main experiment included 30 adult female mice, randomly assigned to one of five groups, six mice each. The first group (Grp 1) received standard care and was injected with saline, whereas other groups (Grps 2–5) were intraperitoneally injected with PTX (GrP 2), Cisplatin (Grp 3), PTX, and 30 µg/kg mdivi-1 (Grp 4) or cisplatin with 30 µg/kg mdivi-1 in DMSO (Grp 5), respectively. Animals in all groups had free access to food and water during the study period. One week after the end of treatment period, animals were euthanized, their ovaries were recovered. For Hematoxylin and Eosin (H&E) staining, ovary tissues were fixed with 4% polyformaldehyde for 24 h, dehydrated, embedded in paraffin and then cut into 5-µm-thick sections. The prepared sections were stained with hematoxylin and eosin (H&E), following the standard protocol, and examined by using light microscope.

### Statistical analysis and software

Data analysis was performed using the SPSS 26.0 software package. All cell culture work was carried out in triplicates. Values of apoptosis, autophagy, and cell cycle were displayed in histograms as a percent of the control and represented as the mean of 3 runs (± SD). Multiple comparison analysis was performed by one way ANOVA test followed by Tukey test. Image J (www. Fiji.com) was employed to retrieve some relevant investigations. *P* values less than 0.5 were considered to indicate significant differences.

## Results

### Drp1 deficiency significantly improved the cytotoxic effect of PTX in PTX-resistant cells

**Figure 1:**
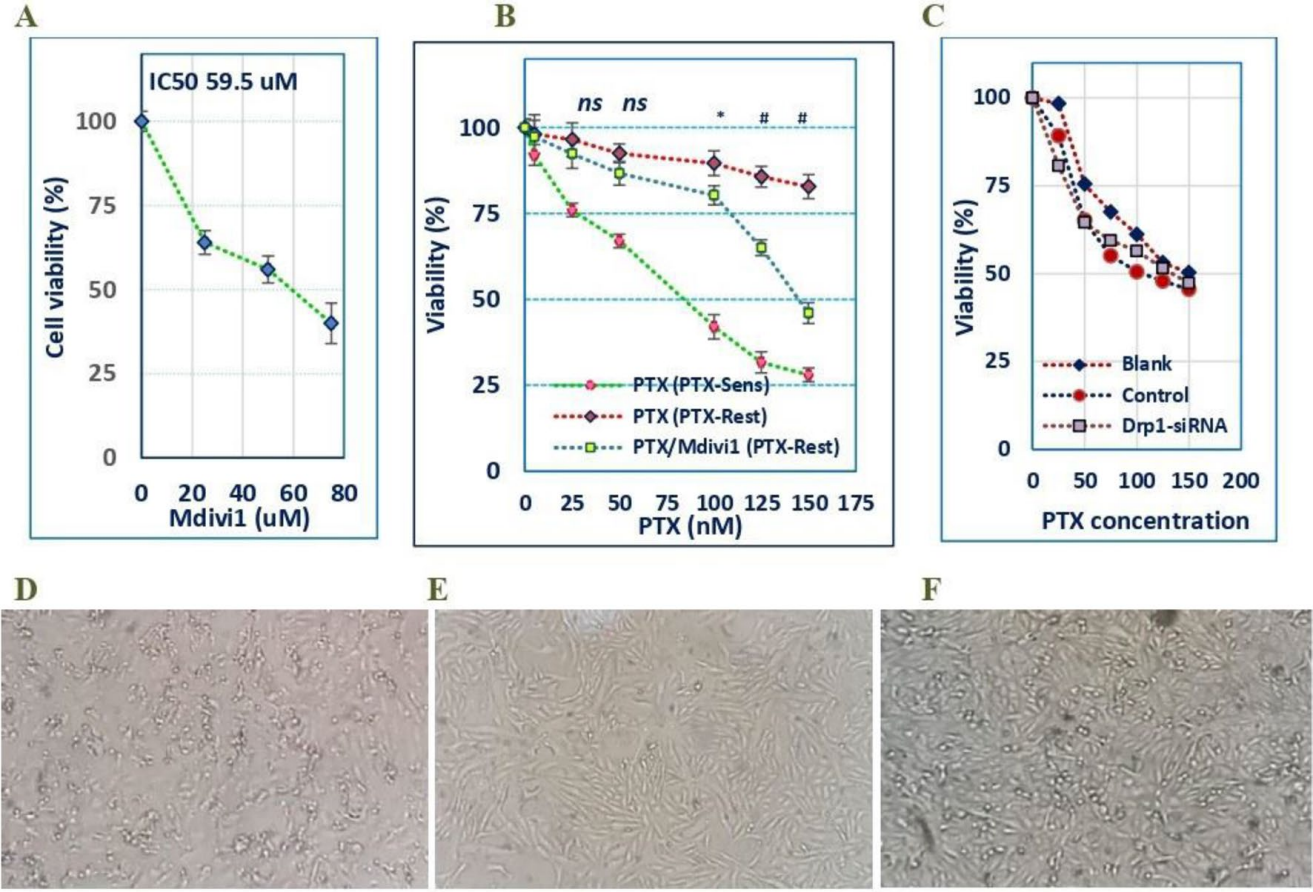
The cytotoxic effect of PTX is enhanced by Drp1 deficiency in breast cancer cells. The cytotoxicity of PTX was evaluated in PTX-sensitive and PTX-resistant cells by MTT assay, where the IC_50_ of mdivi-1 in PTX-sensitive cells was 59.5 µM (**A**). PTX-sensitive cells (green dashed line) and resistant cells (red dashed line) were incubated with a concentration range of PTX. Also, resistant cells were incubated with different concentrations of PTX combined with the IC_50_ of mdivi-1 (blue dashed line) and the percent of viable cells was determined and normalized to the control cells (**B**). Also, the percentage of cell viability was determined in PTX-resistant cells exposed to concentration range of PTX after their transfection Drp1-specific siRNA (**C**). The IC_50_ of PTX in PTX-sensitive cells is 85 nM, where for PTX+mdivi-1 is 132 uM. D through F are representative photomicrographs of PTX-sensative cells treated with PTX, PTX-resistant cells treated with PTX, and PTX-resistant cells breated with both PTX and mdivi-1, respectively (Magnification 20X). Dots represent the mean (± SD) of viable cells of n = 5–6 readings. (*) and (#): refer to significant changes in cell viability of PTX+mdivi-1 versus PTX, *P* < 0.05 and *P* < 0.001, respectively. “D-F” are representative light micrographs of PTX-regular or resistant cells treated with PTX or cells dually treated with PTX+Mdivi1, respectively.

Initially, regular (PTX-sensitive) cells were treated with increasing concentrations of mdivi-1, where the percent of cell viability was evaluated via MTT assay. The obtained IC_50_ concentration (59.5 µM) (Fig. 1A) was applied in the subsequent investigations. Next, we evaluated the cytotoxic effect of PTX, either alone or combined with Drp1-loss in PTX-resistant cells. PTX induced a concentration-dependent cytotoxicity, IC_50_ 85 nM, in PTX-sensitive cells, whereas resistant cells tolerated the same nanomolar concentration range. Combining mdivi-1 with PTX, enhanced the cytotoxic effect, particularly when the resistant cells were treated with higher doses than 100 nM, like 125 and 150 nM PTX, (*P* < 0.05 and *P* < 0.001) (Fig. 1B). The IC_50_ of PTX+mdivi-1 was found to be 132 µM. Lower concentrations (< 100 nM), insignificantly decreased cell viability relative to PTX-treated regular cells (*P* > 0.05). Similarly, resistant cells, pretransfected with Drp1-siRNA followed by PTX treatment demonstrated concentration-dependent cytotoxicity (Fig. 1C). Morphologically, apoptotic features were observed in PTX-treated regular cells, including their shrinkage, rounding, and detachment. Resistant cells, however, displayed similar changes only when they were dually treated with PTX+mdivi-1 (Fig. 1D–F).

### Drp-1 deficiency increased the sensitivity of PTX-resistant cells to apoptosis and autophagy and arrested cells in G2-M phase

**Figure 2:**
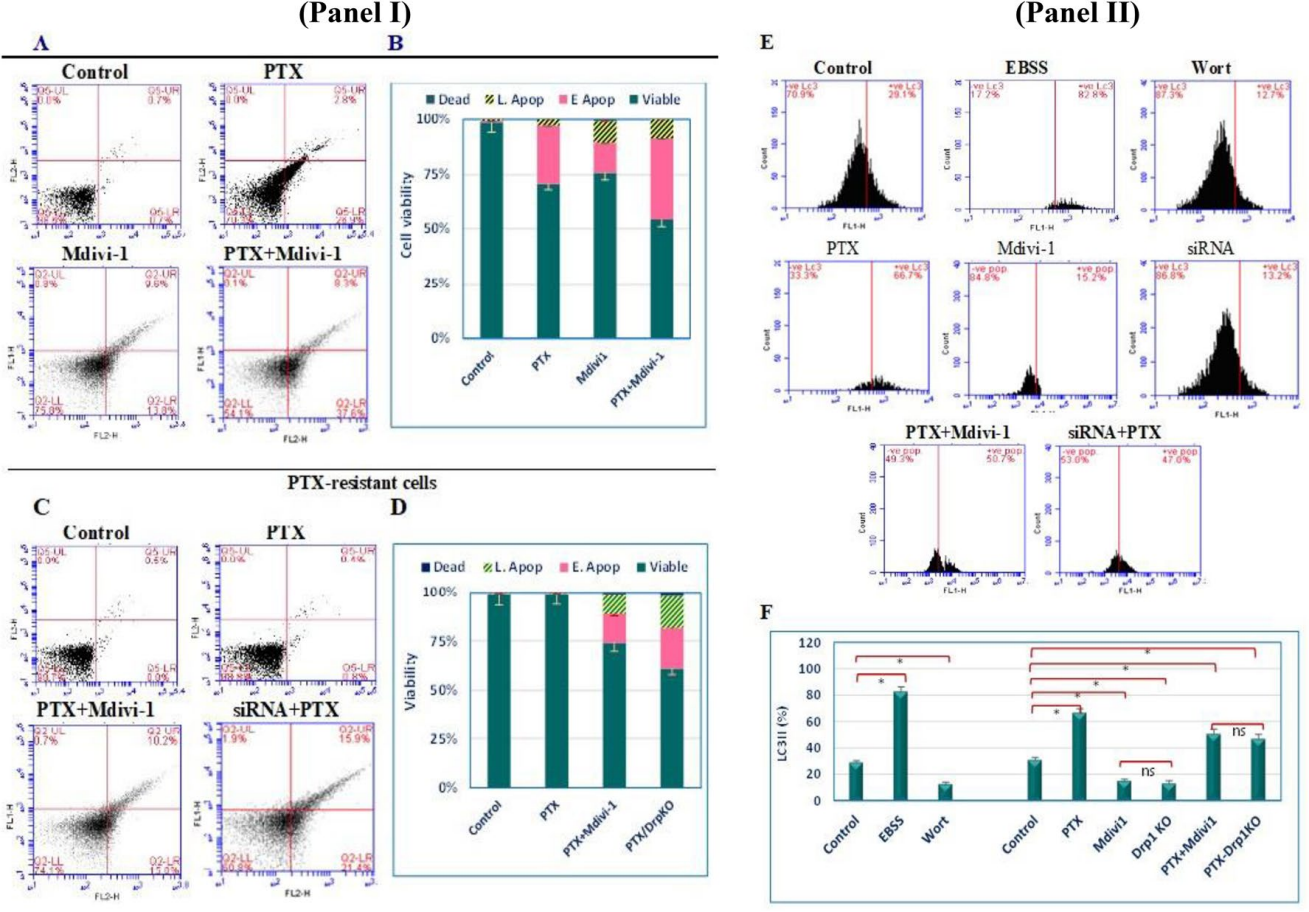
Drp1 deficiency induced apoptosis and autophagy in PTX-resistant cells. Regular cells (**IA**) were left untreated, treated with the IC_50_ concentration (85 nM) of PTX, the IC_50_ concentration (59.5 µM) of mdivi-1, or PTX+mdivi-1. The apoptotic effect in PTX or mdivi-1 treated cells was enhanced in when cells were cotreated with both drugs. Resistant cells (**IC**) were left untreated, treated with PTX, PTX+mdivi-1, or PTX after they were transfected with Drp1-specific siRNA. Apoptosis was significantly developed in PTX cells cotreated with Drp1 inhibitor or transfected with siRNA (**D**). “E” depicts the level autophagy marker (LC3II) in cells left untreated, transiently incubated in EBSS, treated with Wort, PTX, mdivi-1, transfected with siRNA, PTX+mdivi-1, or treated with PTX, after siRNA transfection. Resistant cells responded to the autophagy inducer EBSS or repressor (Wort). PTX induced a twofold increase in the LC3II (*P* < 0.001). Drp1 inhibition (or silencing), insignificantly, repressed autophagy (*P* > 0.05) compared to its basal level. However, combined treatments led to a significant increase in the LC3II. Bars (**B**, **D** and **F**) represent the mean of three independent experiments (± SD), (*): refers to a significant difference between the indicated cells versus the corresponding untreated cells. “ns”: insignificant.

To determine the apoptosis and/or autophagy sensitivity of resistant cells, Annexin-V/PI dual staining and the cellular level of LC3II were evaluated by flow cytometry. PTX, mdivi-1, and PTX+mdivi-1 developed apoptosis in 29.7%, 24.4%, and 45.9% of the regular cells, respectively. Furthermore, PTX did not induce considerable apoptosis in PTX-resistant cells; however, the dual treatment led to apoptosis in 25.2% and 37.3% of cells, when they were cotreated with PTX+mdivi-1 or pretransfected with Drp1-specific siRNA then exposed to PTX, respectively (Fig. 2I). To assess the corresponding autophagy, resistant cells were transiently incubated in EBSS or treated with Wort (100 nM), to induce or inhibit autophagy, as positive and negative controls, respectively. The changes in LC3II protein indicated the responsiveness of the resistant cells to autophagy modulators, where LC3II levels in cells, grown in EBSS or treated with Wort, were 82.8 ± 3.4% and 12.7 ± 1.1%, respectively (Fig. 2II). Also, we observed that the loss of the Drp1 reduced LC3II in mdivi-1-treated cells (13.8 ± 0.9%) or siRNA pretransfected cells (13.2 ± 2%). Additionally, exposure of cells to PTX, mdivi-1+PTX, or PTX after transfection with siRNA, increased the level of LC3II relative to its basal measurements in DMSO-treated cells (66.7 ± 2.8%, 50.7 ± 3.5%, 47 ± 3.1%, respectively). Cell cycle analyses revealed that PTX and Mdiv-1 arrested 49.7% and 43.11% of cells in G2-M phase, respectively, compared to 13.8% in DMSO-treated cells (*P* < 0.001, *P* < 0.001, respectively). Also, both drugs significantly and independently reduced the number of cells in the synthesis (S) phase. However, combined treatments did not show synergistic nor additive effects (Table 2).

**Table 2:** Distribution of cell cycle phases in breast cancer cells left untreated, treated with PTX, mdivi-1, PTX combined with mdivi-1, or PTX after cell transfection with Drp1-specific siRNA.

Phase	Control	PTX	Mdivi-1	PTX + Mdivi-1	PTX + Drp1KO
G0-G1	28.41 ± 1.7	22.32 ± 1.1 **	19.5 ± 1.3 ***	24.68 ± 2.2 ns	20.68 ± 1.5 **
S	57.04 ± 3.3	26.99 ± 1.5 ***	36.5 ± 1.8 ***	26.73 ± 1.2 ***	38.36 ± 2.3 ***
G2-M	13.7 ± 1.1	49.68 ± 3.5 ***	43.11 ± 2.9 ***	47.55 ± 3.7 ***	40.07 ± 3.6 ***

(*): comparison of the indicated group versus control; (#): compared to PTX; ns: none significant.

### Drp1 deficiency upregulated apoptosis-related genes, downregulated cell cycle-related genes, and MDR1, and relieved the cellular oxidative stress.

**Figure 3:**
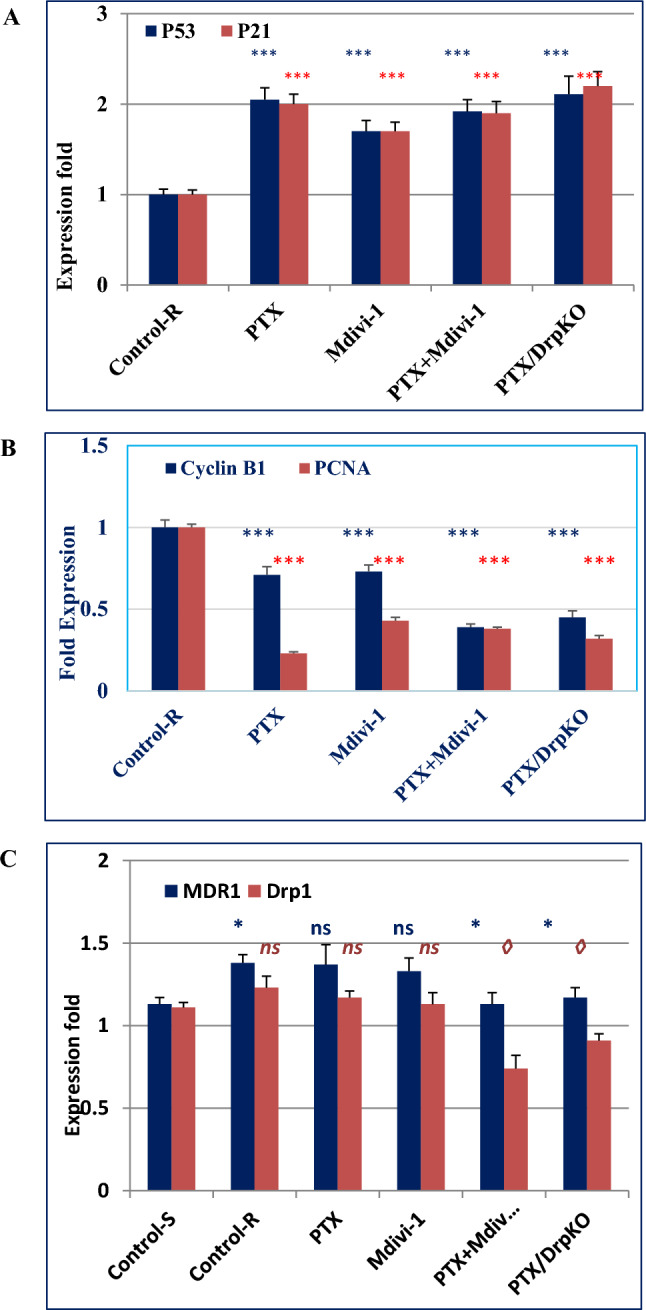
Expression of apoptosis-related genes (p21 and p53), cell cycle-related genes (cyclin B1 and PCNA1), MDR1, and Drp1. RNAs were isolated form untreated or treated cells, reverse transcribed, and then used as templates in qRT-PCR reactions. In PTX-resistant cells, the expression of p53 and p21 increased (**A**), meanwhile cyclin B1 and PCNA1 were downregulated (**B**) relative to the untreated resistant cells. MDR1 expression was higher in resistant cells compared to sensitive cells (Control-S), and decreased in dually-treated resistant cells compared to the corresponding untreated cells. No significant difference was seen between normal and resistant cells; however it decreased in dully treated resistant cells.

**Figure 4:**
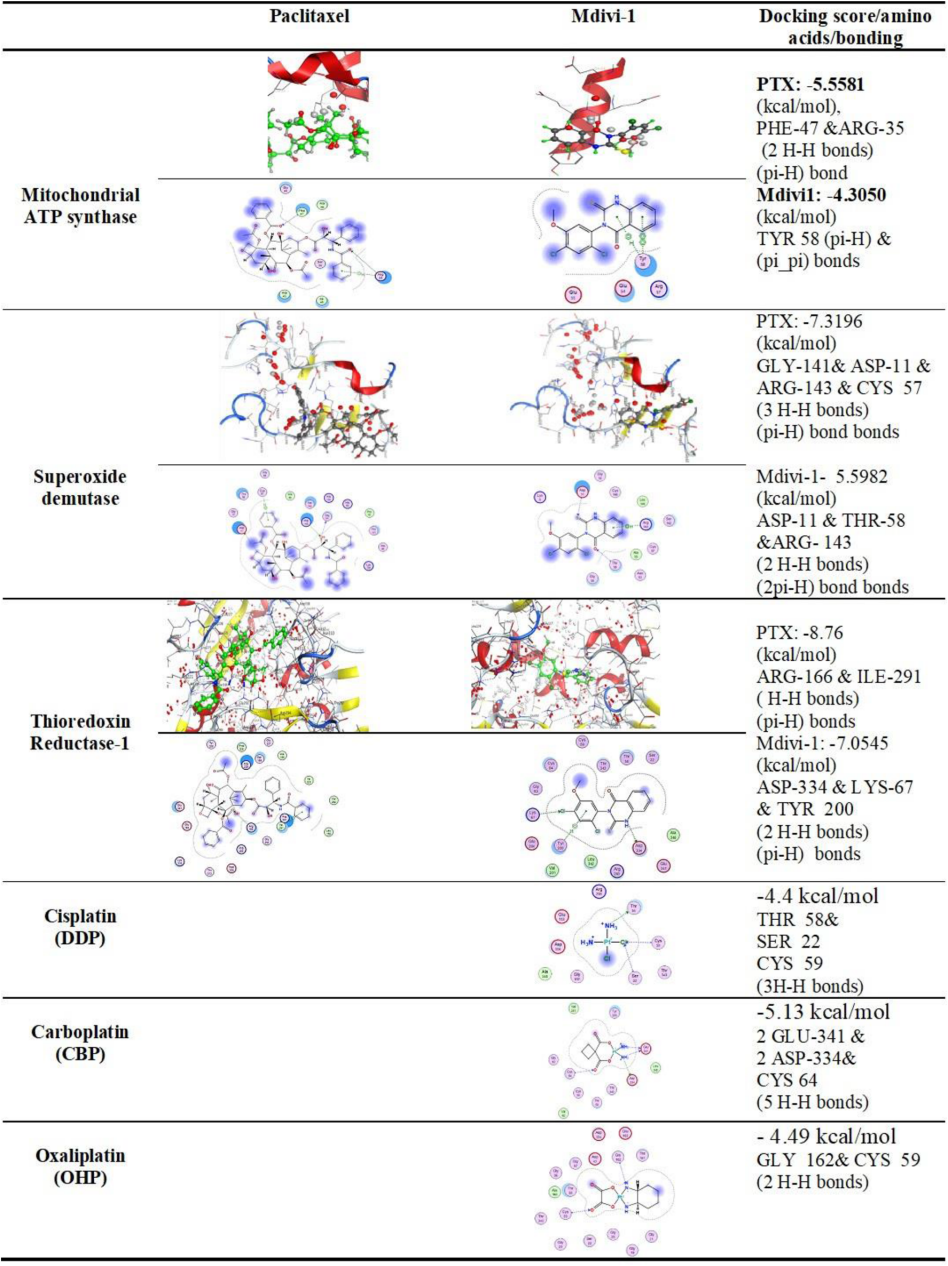
Computational modeling of the molecular interactions of paclitaxel, and mdivi-1 with mitochondrial redox-related enzymes. The 2D interaction diagrams demonstrate the interaction of ligands (PTX or mdivi-1) with mitochondrial ATP synthase, SOD or Thioredoxin reductase 1. The hot spots in protein domains, the superimposition of the docking pose, and the co-crystallized ligands in the active sites in the enzyme active site are shown. The docking score is shown next each protein.

Expression analyses of apoptosis-related genes and G2-M cell cycle-specific genes, at the mRNA level, demonstrated that p53 and p21 genes were upregulated compared their basal level in PTX-resistant cells. Also, cyclin B1 and the proliferating cellular nuclear antigen (PCNA1) were downregulated following combination treatments. No sysnergestic effect was observed in cells cotreated with both drugs (Fig. 3A,B). Resistant cells demonstrated higher expression of MDR1 (*P* < 0.05), relative to regular cells. Moreover, dual treatments (with mdivi-1 or siRNA), similarly reduced its expression (*P* < 0.05) (Fig. 3C). In parallel, Drp1 expression did not show a significant difference between regular and sensitive cells (*P* > 0.05) however, it decreased in dully treated resistant cells, relative to the untreated one (*P* < 0.05, *P* < 0.05). In all expression analysis, mdivi-1 did not show significant difference compared to siRNA-mediated depletion. As mitochondrial functions depend on redox reactions, it was important to examine the associated ROS-related changes. As Table 3 shows, resistant cells have higher H2O2 (*P* < 0.05) and less SOD (*P* < 0.001) than regular cells. PTX alone enhanced the production of H2O2 but mdivi-1, alone or combined with PTX, significantly improved the level of both markers. Computational analysis suggested molecular interactions of PTX and mdivi-1 with SOD and other mitochondrial enzymes ATP synthase and Thioredoxin reductase 1 (Fig. 4).

**Table 3:** Changes in hydrogen peroxide level and the activity of SOD in PTX-sensitive cells and PTX-resistant cells cotreated with Drp1 inhibitors.

	Control-S	Control-R	PTX-R	Mdivi1-R	PTX + Mdivi1-R	PTX/Drp1KO-R
H2O2	9.19 ± 0.77	11.73 ± 0.86 * ns	12.96 ± 0.56 *** ns	9.05 ± 0.75 ns # ◊◊◊	9.36 ± 0.96 ns # ◊◊	9.42 ± 0.85 ns # ◊◊
SOD	113.11 ± 8.94	77.83 ± 4.73 *** ns	85.63 ± 5.77 ** ns	106.66 ± 8.72 ns ## ◊	101.67 ± 5.48 ns ##	100.62 ± 5.71 ns #

Averages were generated from 3 independent experiments for each mouse-derived blood samples.

(*): significant difference compared to the untreated sensitive cells.

(#): *P* < 0.05 significant difference compared to the untreated resistant cells.

(^◊^) significant difference compared to PTX.

### Drp1 deficiency promoted the expression of the mitochondrial fusion proteins, modulated the mitochondrial morphology, and restricted cell migration.

**Figure 5:**
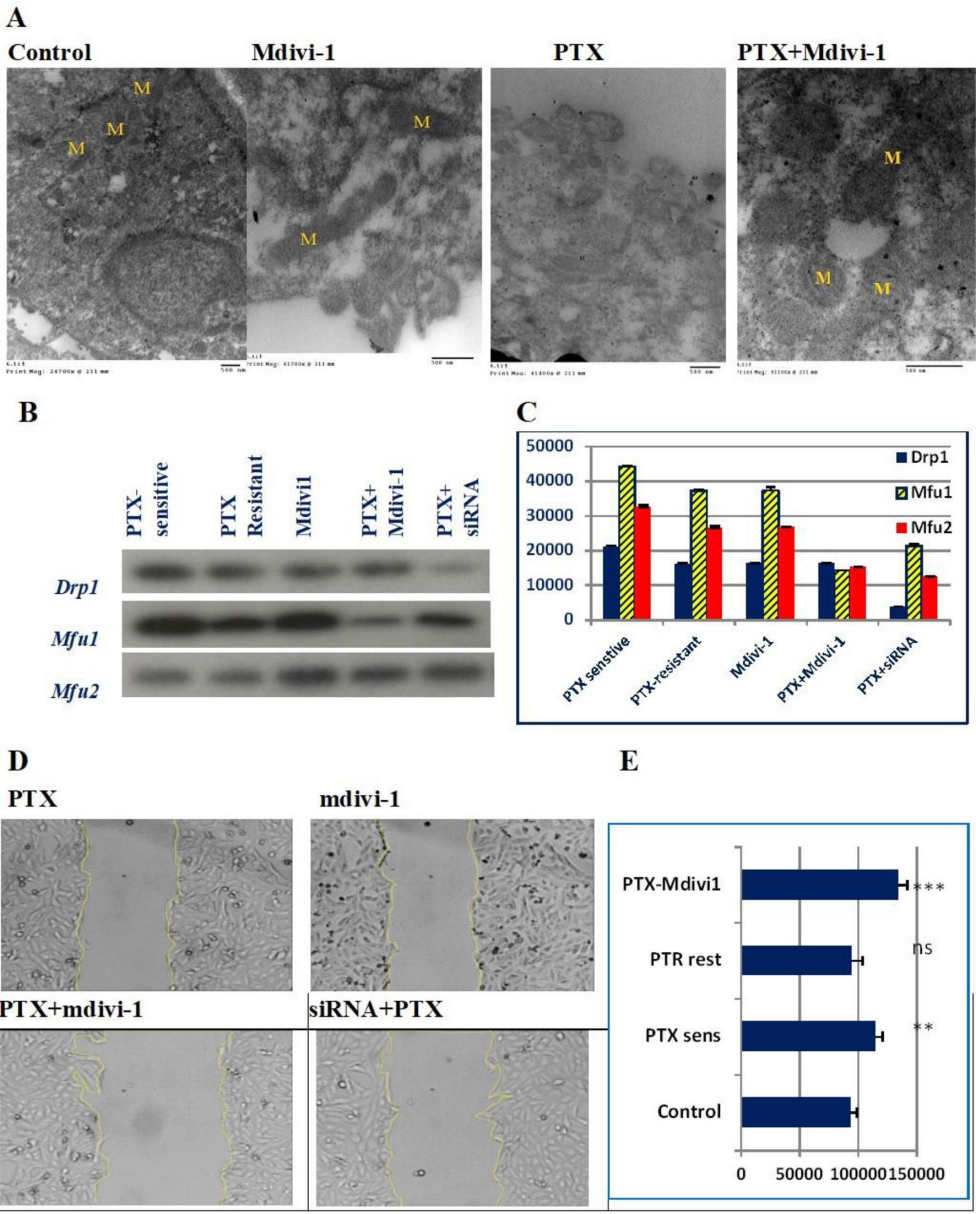
Changes in the mitochondrial morphology, and mitochondrial dynamic-related proteins. Electronmicrographs of the mitochondrial morphological changes in PTX-resistant cells (**A**), cells treated with mdivi-1, PTX, or PTX+mdivi-1. (**B**) depicts immunoblotting of the mitochondrial-related protein (Drp1), and fusion proteins (Mfu1 & Mfu2) and the corresponding band intensities (**C**). Drp1 expression was normally expressed in PTX-resistant cells, similar to regular cells. Mdivi-1, alone or combined with PTX did not affect the expression of Drp1, whereas it is downregulated in cells treated with PTX after siRNA transfection. Mfu1&Mfu2 were significantly upregulated in cell treated with mdivi-1 or PTX after siRNA transfection. (**D**) shows the in vitro cell migration of cells treated with PTX, Mdivi-1, mdivi-1+PTX, or cells transfected with siRNA and then were treated with PTX. *, **, *** refer to *P* < 0.05, *P* < 0.01, *P* < 0.001, compared to the untreated cells.

To determine the associated mitochondrial morphological changes, cells were fixed, stained, and imaged by TEM. The electromicrographs showed condensed mitochondrial in resistant cells. However, mitochondrial morphological alterations, elongation, or enlargement, have been detected when cells were treated with PTX, mdivi-1, or both (Fig. 5A). To explore the intervening factors for the mitochondrial changes, the abundance of the mitochondrial dynamic-related proteins (Drp1, Mfu1, and Mfu2) was determined. We found that the expression of fusion proteins (Mfu1/2) was higher than fission protein (Drp1), when cells were treated with Mdvi-1, PTX combined with mdivi-1, or PTX after cells have been transfected with Drp1-specific siRNA. Notably, Drp1 was effectively inhibited as a result of siRNA than mdivi-1 (Fig. 5B,C), meanwhile mdivi-1 did not show significant downregulation in Drp1 expression. These cellular, molecular, and biochemical changes were associated with the inhibition of in vitro cell migration (Fig. 5D,E).

### Drp1 deficiency did not restore the ovarian hormones and the normal follicular count in PTX-nor cisplatin intoxicated mice

**Figure 6:**
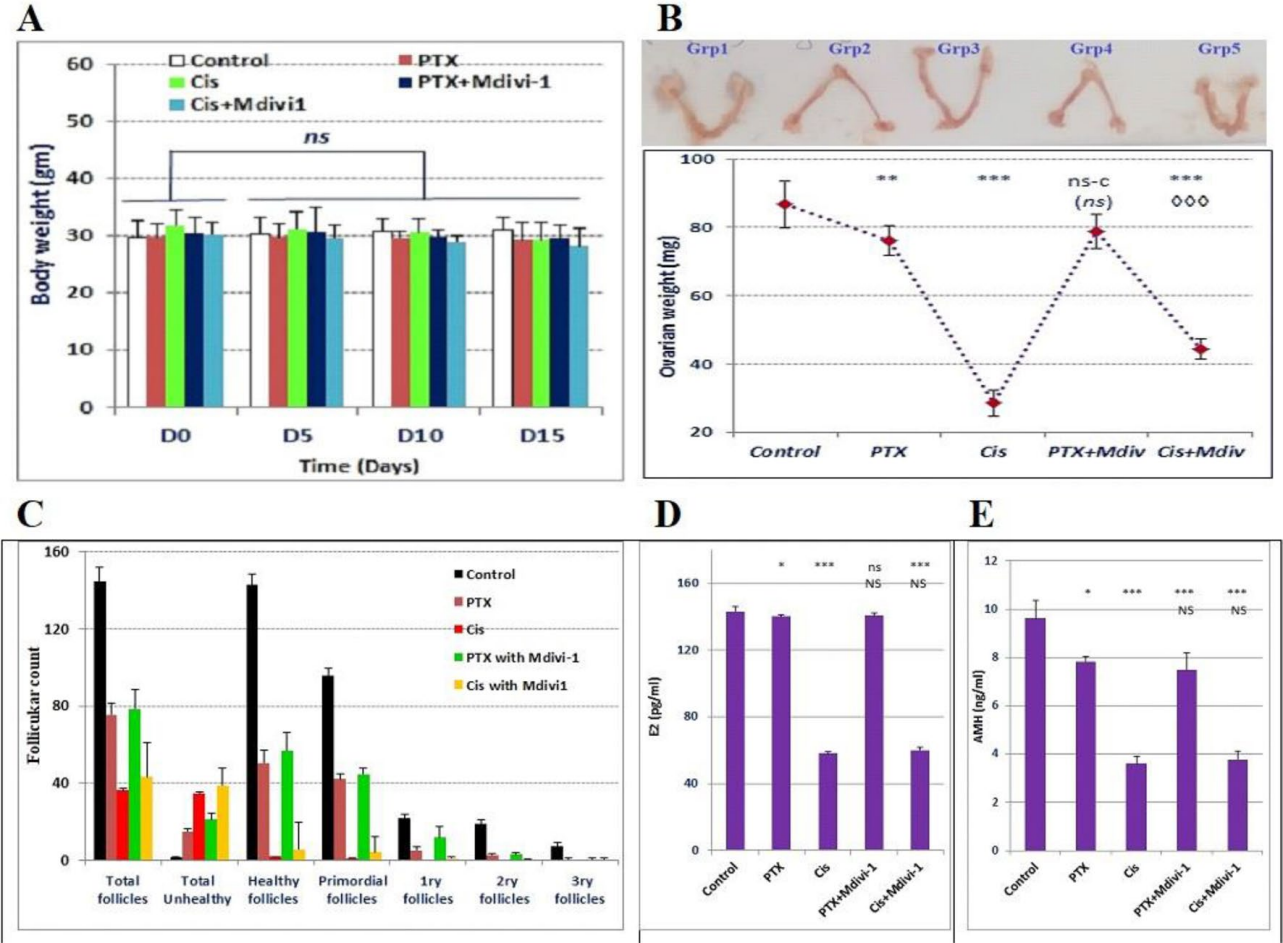
Change in mice body weight, ovarian weight, follicular count, and E2 and AMH levels. Mice body weight (**A**) after various treatments were compared to the initial body weight (Day 0), ovarian weights of treated groups were compared to the corresponding control group (**B**). Differential follicular count demonstrated massive decrease of the count of various types (**C**). Both E2 (**D**) and AMH (**E**) decreased in PTX or cisplatin groups, whereas cotreatment with mdivi-1 did not show significant improvements compared to PTX or cisplatin groups. Data are shown as bars or dots indicating the mean (± SD). *Abbreviations*: E2: estradiol, AMH: antimullerian hormone.

**Figure 7:**
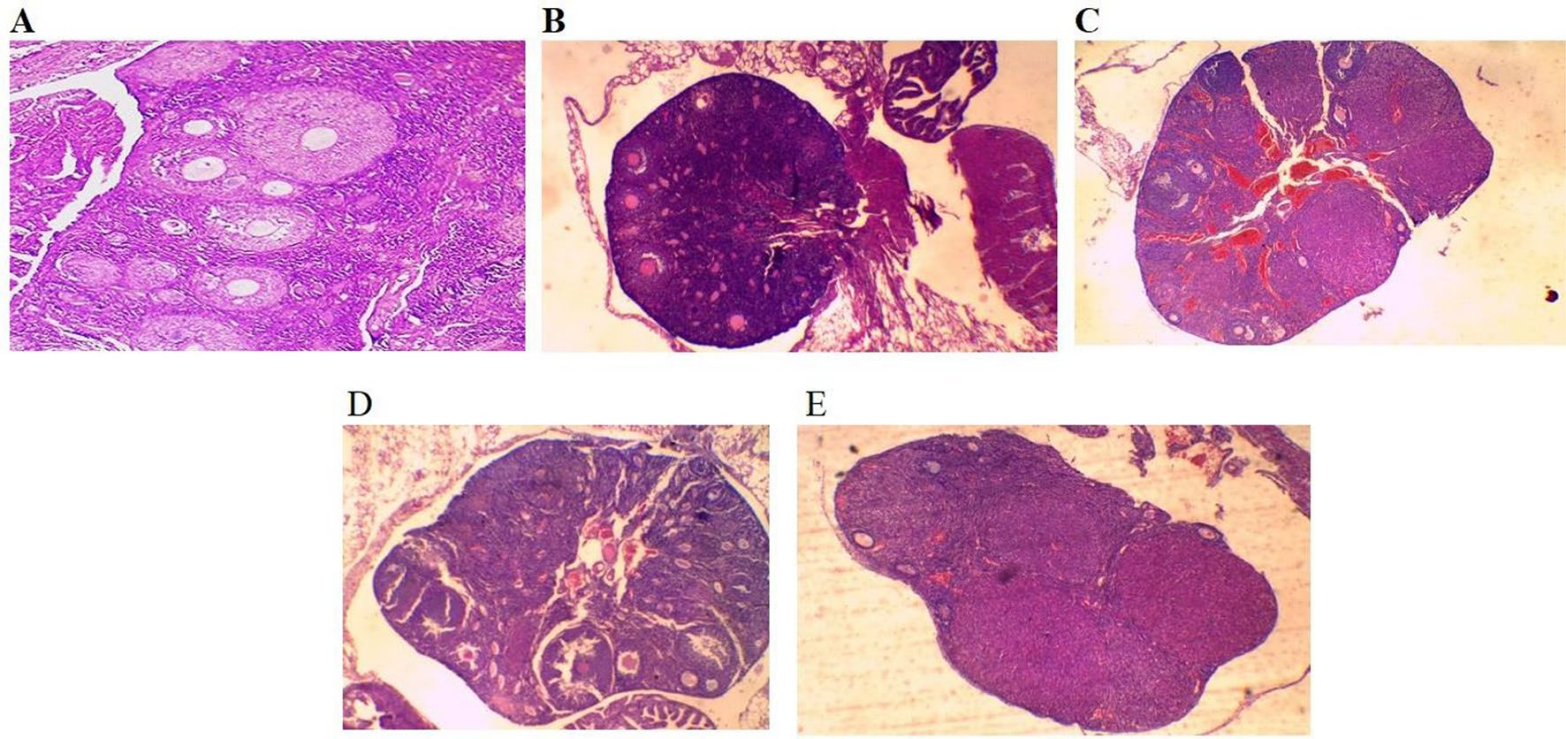
Representative hematoxylin and eosin-stained ovarian sections recovered from healthy mice (**A**), mice intraperitoneally injected with PTX (**B**), Cisplatin (**C**), PTX+mdivi-1 (**D**), or Cis+mdivi-1 (**E**). Both PTX and Cisplatin reduced the size of ovaries, the number of healthy follicles. Cotreatment with mdivi-1did not exert a significant repair.

Chemotherapy is known to induce follicles depletion and subsequent infertility. It was imperative to monitor the gonadotoxic effect of repetitive doses of PTX and evaluate the possible protective role of mdivi-1 in mice compared to cisplatin-induced POF. We observed an insignificant decrease in body weight of PTX- and Cisplatin-intoxicated animals (Grp2&Grp3), relative to their initial body weight (Fig. 6A). Regarding the changes in the ovaries, several observations were found. First: PTX moderately (*P* < 0.01) reduced the ovary weight, compared to cisplatin (*P* < 0.001); Second: no significant difference was seen in the ovary weight between PTX-intoxicated group (Grp2) and PTX/Mdivi-1 treated group (Grp4) (*P* > 0.05). The improvement observed in Cis/mdivi-1 (Grp5) was insufficient to restore normal ovarian weight (*P* < 0.001). Similarly, PTX/Mdivi1 treatment did not induce significant improvement in ovarian weight (Fig. 6B). The count of healthy follicles decreased in PTX-treated mice and severely decreased in the cisplatin-intoxicated group. The differential follicular count (Fig. 6C) did not show significant improvement when animals were treated with PTX/mdivi-1 or Cis/mdivi-1. Also, treated groups did not restore the normal levels of E2 and AMH (Fig. 6D,E). Histologically, the normal control group demonstrated healthy primordial, primary, secondary, and tertiary follicles and no signs of apoptosis in the granulosa cells. However, mice intoxicated with PTX or Cisplatin revealed marked deteriorations including unhealthy oocytes, apoptosis of granulosa, and high reduction in secondary and tertiary follicles numbers. Moreover, combined treatment with mdivi-1 did not generate significant healing signs (Fig. 7).

## Discussion

Drug-resistance and chemotherapy-associated ovarian failure are among the main characteristics of triple-negative breast cancer, where they represent the primary etiology of treatment failure, recurrence, and chemotherapy-related infertility in breast cancer patients^21^. PTX is commonly used as a first-line treatment in breast cancer; however, its implication in premature ovarian insufficiency in young women was inadequately explored^22^. This study demonstrated that the development of drug resistance in TNBC and the associated Drp1 deficiency revealed some molecular and cellular observations. First: PTX concentration (nanomolar or micromolar) differently affected cell viability and the cell death mode of PTX-resistant cells versus regular cells. Second: In agreement with previous studies^23^, PTX resistance may involve the upregulation of drug efflux-related genes, where PTX-resistant cells were overexpressing MDR1, meanwhile Drp1 inhibition mildly reduced the expression of drug efflux-related gene. Third: The development of drug resistance did not affect the expression of Drp1, accordingly the effect of mdivi-1 or Drp1-specific siRNA on resistant cells was not interrupted by intervening factors. Fourth: The adaptability of breast cancer cells to drug resistance involved the modulation of their redox-related machinery^24^ to be accommodated with the observed overproduction of H2O2 and decrease the activity of ROS-scavenging enzymes (SOD). Previous studies suggested that mdivi-1 induces DNA replication stress, as it damages DNA, impairs DNA replication, represses mitochondrial respiration, and triggers mitochondrial uncoupling^13^ and swelling as we observed in mdivi-1 treated cell. Fifth: Resistant cells were responsive to autophagy modulators, where both glucose oxygen deprivation (GOD) and Wort were oppositely affected the level of LC3II protein. Depite the complexity of drug-resistance, these fundamental observations largely shaped the sensitizing effect of Drp1 deficiency. Although Drp1 is the key player in the mitochondrial severing process, it participates in other cellular activities including apoptosis-mediated cell death^25^. Cell viability studies revealed that Drp1 inhibition enhanced the responsiveness of resistant cells to PTX, especially when they were cotreated with 100 nM PTX or more. Micromolar concentrations (50 µM), however, led to massive necrosis of the PTX-sensitive cells when they were exposed to PTX alone or combined with Drp1 inhibitor (*data not shown*). This emphasizes the biphasic cytotoxic effect of PTX^26^. In the same context, and similar to previous studies^27^, Drp1 inhibition led to the development of apoptosis in about one-fourth of cells. Also, Drp1 inhibition synergistically supported PTX-mediated apoptotic effect in resistant cells, where 25.5 or 37.3% of cells developed apoptosis as a result of combining PTX with mdivi-1 or siRNA transfection, respectively. This may be attributed to the accumulation of Bcl-2-associated X protein (BAX) at mitochondrial fission sites that promoted the permeabilization of the outer mitochondrial membrane and cytochrome *c* release^28^. Alternatively, changes in Drp1 may derive the balance of the mitochondrial dynamics (fission and fusion), where increased the expression of fusion proteins (Mfu1/2) and mitochondrial elongation and enlargements were detected in PTX-resistant cells, treated with mdivi-1 alone or combined with PTX. Other investigators stated that Drp1 modulation may involve mitochondrial Ca^2+^ responses in apoptotic signaling^29^ and caspase-3 activation^30^. Additionally, building up ROS may represent a key factor in the overproduction of fusion proteins (Mfu1/2) that shifted the mitochondrial dynamics towards fusion. Mechanistically, mdivi-1 inhibits the GTP-induced Drp1 conformational changes that are required for Drp1 self-assembly^31^, and subsequent mitochondrial hyperfusion and elongation^32,33^. The observed mdivi-1-mediated apoptosis seems to be cell-type dependent as in renal cell carcinoma the drug wasn’t able to exert apoptotic effects, but it sensitized cells to cisplatin^34^. Our findings do not exclude the direct impact of mdivi-1 on different mitochondrial redox-related and energy-related enzymes, as the brief computational analysis we performed predicted the direct inhibitory effects of mdivi-1 on mitochondrial enzymatic machinery including ATPase. In addition to apoptosis, autophagy seems to be an alternative cell death mode. In agreement with previous studies^35^, PTX caused a twofold increase in the autophagy marker, where it is regarded as an adaptive mechanism utilized by resistant cells against PTX-mediated caspase-dependent apoptotic cell death^36,37^. The Drp1-mediated apoptotic and autophagic events we observed were associated with G2/M cell cycle arrest and downregulation of G2/M-specific genes like PCNA and cyclin B1. This may be attributed to the direct involvement of Drp1 deficiency in ATM-dependent DNA replication stress^38^.

In another context, recent studies reported that PTX monotherapy was associated with a strong gonadotoxic effect in premenopausal patients^39,40^. Accordingly, it was important to investigate how far mdivi-1 can repair PTX-associated ovarian damage. We found that cisplatin-induced more severe gonadotoxic effect and deteriorated the blood levels of both E2 and AMH, relative to the corresponding effect of PTX, when they were taken in similar repetitive doses. This may be explained by the diverse mechanisms adopted by each drug. Evidence generated from animal models suggested that PTX-induced ovarian damage is mediated by abnormal cell division, oxidative stress, and caspase-dependent apoptosis^41,42^. Obviously, the latter was massively detected in the granulosa cells in mice intoxicated either with PTX or Cisplatin. The apoptotic effect of Cisplatin is caused by its direct interaction with cell’s DNA, formation of adducts that eventually leads to DNA damage and cell death (reviewed by Dasari and Tchounwou^43^). Although a long list of preclinical and clinical studies were undertaken to maintain ovarian integrity during chemotherapeutic administration, none of these studies presented Drp1 deficiency as a potential protective tool. Based upon its role in attenuating oxidative metabolism in cancer cells^15^, and its potential in resensitizing breast cancer cells, we outlined above, mdivi-1 is a potential protective agent to attenuate chemotherapy-derived POF. Although it didn’t provide satisfactory outcomes, the slight improvement in the follicular count may predispose to more investigations to reoptimize its dose, mode, and duration of admonition.

## Conclusion

In summary, although the concept of combining Drp1 deficiency with traditional chemotherapy was previously suggested, this work demonstrated the interplay between Drp1 inhibition, the alterations in mitochondrial dynamics-related proteins, and the morphological distortion. This led to a significant improvement in the responsiveness of breast cancer cells to PTX. Also, the associated changes in ROS-related markers, the computational modeling, may suggest the direct effect of mdivi-1 and PTX on the mitochondrial enzymes. In parallel, the study investigated, for the first time, the gonadotoxic effect of PTX and the potential of Drp1 inhibitor (mdivi-1) in protecting the ovarian integrity against PTX-induced ovarian insufficiency. As the mitochondrial role in drug resistance may involve metabolic and apoptotic regulatory mechanisms, more investigations are needed to explore the side Drp1-indepenednt talks of mdivi-1 that affect cell survival. Also, as drug resistance in cancer cell is closely associated with the dynamic regulation of mitochondrial dynamics and metabolism, more invetigations are required to explore the associated changes in the mitochondrial metabolism related genes. Additionally, more in vivo investigations are required to explore the direct effect of mdivi-1 on ovarian-related developmental factors and the associated endocrine outcomes.

### Supplementary Information

Supplementary Information.

## Data Availability

The datasets generated during the current study are available at https://zenodo.org/records/10042053.
