# Author Correction: Inhibition of Drp1 orchestrates the responsiveness of breast cancer cells to paclitaxel but insignificantly relieves paclitaxel‑related ovarian damage in mice

**DOI:** 10.1038/s41598-024-60556-y

**Published:** 2024-04-29

**Authors:** Adel I. Alalawy, Mohamed Sakran, Fahad M. Alzuaibr, Maeidh A. Alotaibi, Mohamed E. El‑Hefnawy, Abdulelah Y. Hazazi, Saad M. El‑Gendy, Esraa A. Aidy, Heba Effat, Doha F. Ismail, Mohamed Hessien

**Affiliations:** 1 Department of Biochemistry, Faculty of Science, University of Tabuk, 71491 Tabuk, Saudi Arabia; 2 Division of Biochemistry, Faculty of Science, Tanta University, Tanta City, 31512 Egypt; 3 Biology Department, Faculty of Science, Tabuk University, Tabuk, Saudi Arabia; 4 King Faisal Medical Complex Laboratory, Ministry of Health, Taif, Saudi Arabia; 5 Department of Chemistry, Rabigh College of Sciences and Arts, King Abdulaziz University, Jeddah, Saudi Arabia; 6 Department of Cancer Biology, National Cancer Institute, Cairo University, Giza, Egypt; 7 Molecular Cell Biology Unit, Division of Biochemistry, Department of Chemistry, Faculty of Science, Tanta University, Tanta, 31512 Egypt

Correction to: *Scientific Reports* 10.1038/s41598-023-49578-0, published online 20 December 2023

The original version of this Article contained an error in the spelling of the author Heba Effat which was incorrectly given as Heba E. Abdelaziz.

The original Article has been corrected.

