# Supplementary material for: Inhibition of Drp1 orchestrates the responsiveness of breast cancer cells to paclitaxel but insignificantly relieves paclitaxel-related ovarian damage in mice
Source: Sci Rep. 2023 Dec 20;13:22782. doi: 10.1038/s41598-023-49578-0 (PMC10739747; doi:10.1038/s41598-023-49578-0)
Supplement: Supplementary file 1 — Supplementary Information. [file 41598_2023_49578_MOESM1_ESM.pdf]

Original and Supplementary materials

Inhibition of Drp1 orchestrates the responsiveness of breast cancer cells to paclitaxel *but insignificantly relieves paclitaxel-related ovarian damage in mice*

and original data

Figure 1

A B C

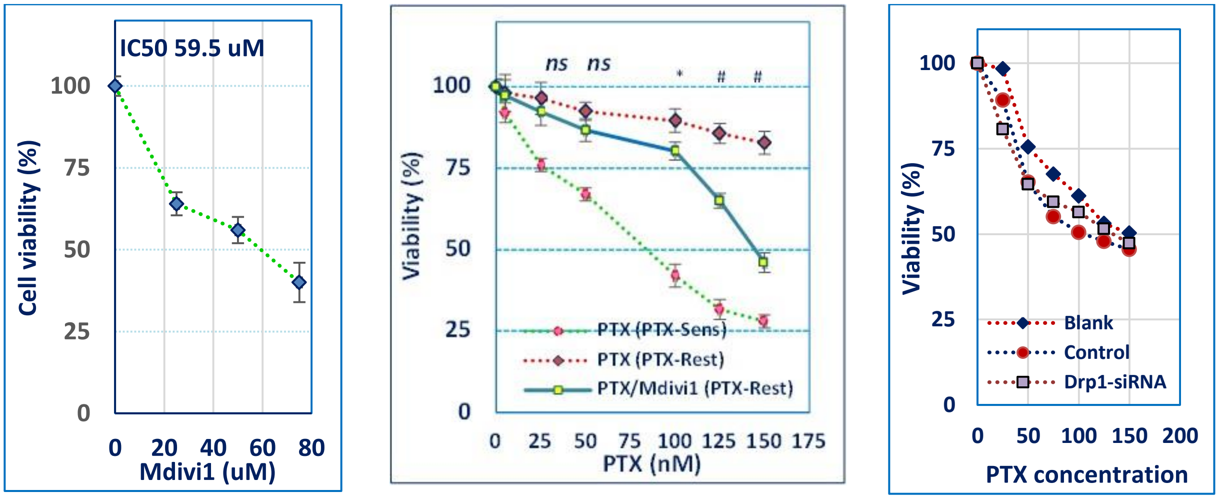

D E F

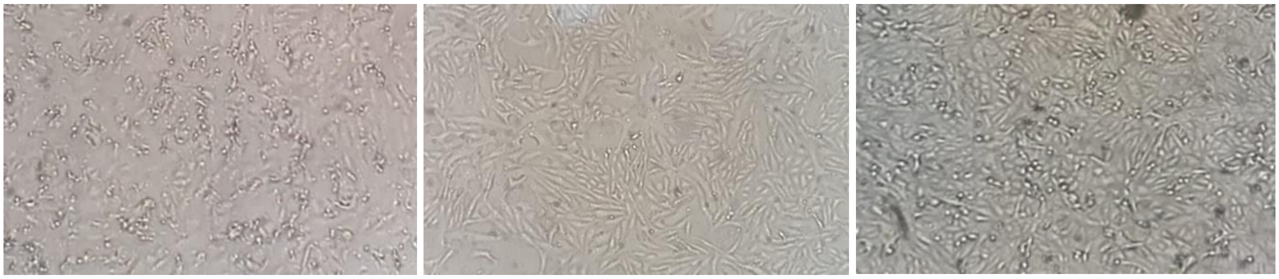

Magnification (20X) Magnification (20X) Magnification (20X)

Data Steps Window Help

Ordering InStat

Comparison	Difference	q		P value
5ptx vs 25ptx	4.900	2.635	ns	P>0.05
5ptx vs 50PTX	10.600	5.699	**	P<0.01
5ptx vs 100ptx	17.000	9.140	***	P<0.001
5ptx vs 5both	-0.8000	0.4301	ns	P>0.05
5ptx vs 25both	0.8000	0.4301	ns	P>0.05
5ptx vs 50both	4.800	2.581	ns	P>0.05
5ptx vs 100both	7.700	4.140	ns	P>0.05
25ptx vs 50PTX	5.700	3.065	ns	P>0.05
25ptx vs 100ptx	12.100	6.506	**	P<0.01
25ptx vs 5both	-5.700	3.065	ns	P>0.05
25ptx vs 25both	-4.100	2.204	ns	P>0.05
25ptx vs 50both	-0.1000	0.05377	ns	P>0.05
25ptx vs 100both	2.800	1.505	ns	P>0.05
50PTX vs 100ptx	6.400	3.441	ns	P>0.05
50PTX vs 5both	-11.400	6.129	**	P<0.01
50PTX vs 25both	-9.800	5.269	*	P<0.05
50PTX vs 50both	-5.800	3.118	ns	P>0.05
50PTX vs 100both	-2.900	1.559	ns	P>0.05
100ptx vs 5both	-17.800	9.570	***	P<0.001
100ptx vs 25both	-16.200	8.710	***	P<0.001
100ptx vs 50both	-12.200	6.559	**	P<0.01
100ptx vs 100both	-9.300	5.000	*	P<0.05
5both vs 25both	1.600	0.8603	ns	P>0.05
5both vs 50both	5.600	3.011	ns	P>0.05
5both vs 100both	8.500	4.570	ns	P>0.05
25both vs 50both	4.000	2.151	ns	P>0.05
25both vs 100both	6.900	3.710	ns	P>0.05
50both vs 100both	2.900	1.559	ns	P>0.05

Figure 2 (Panel I)

Paclitaxel sensitive cells

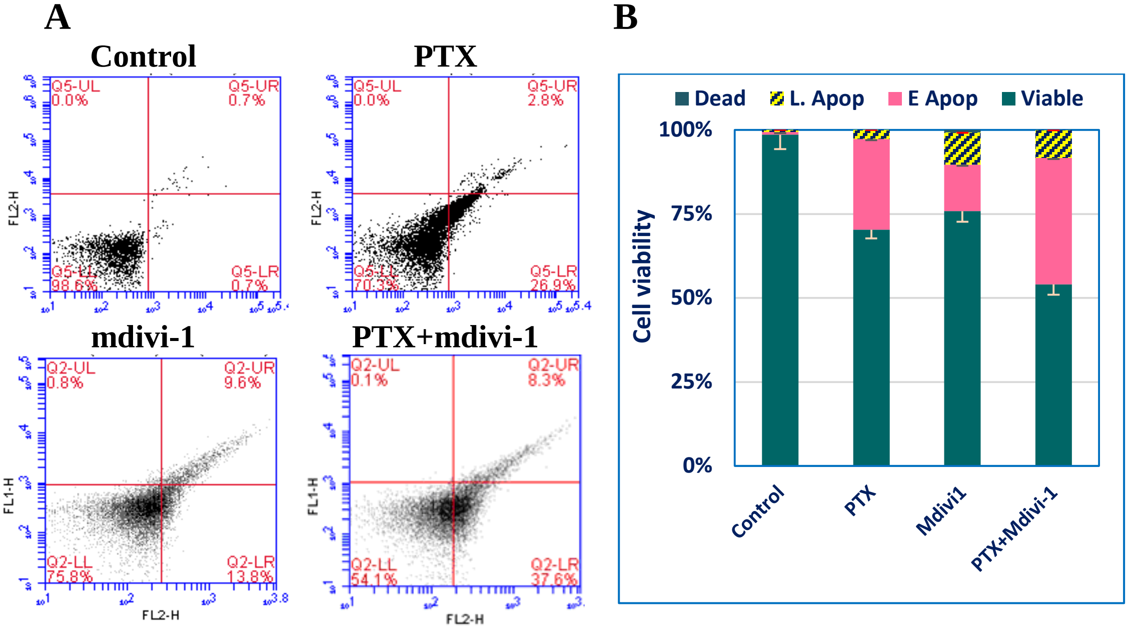

PTX-resistant cells

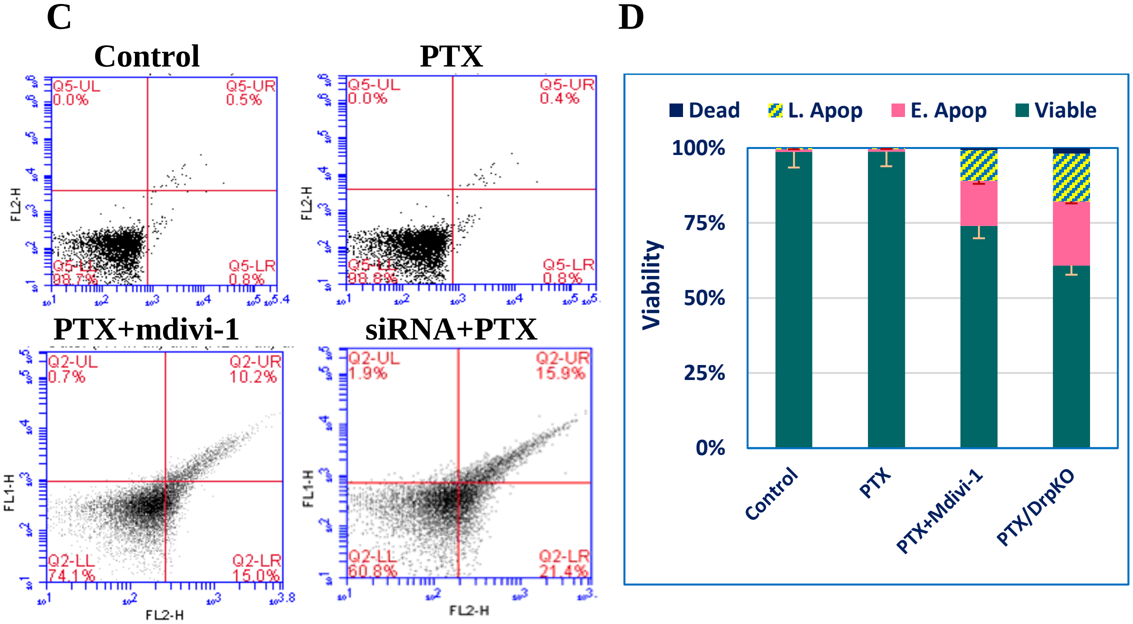

Figure 2 (II)

E

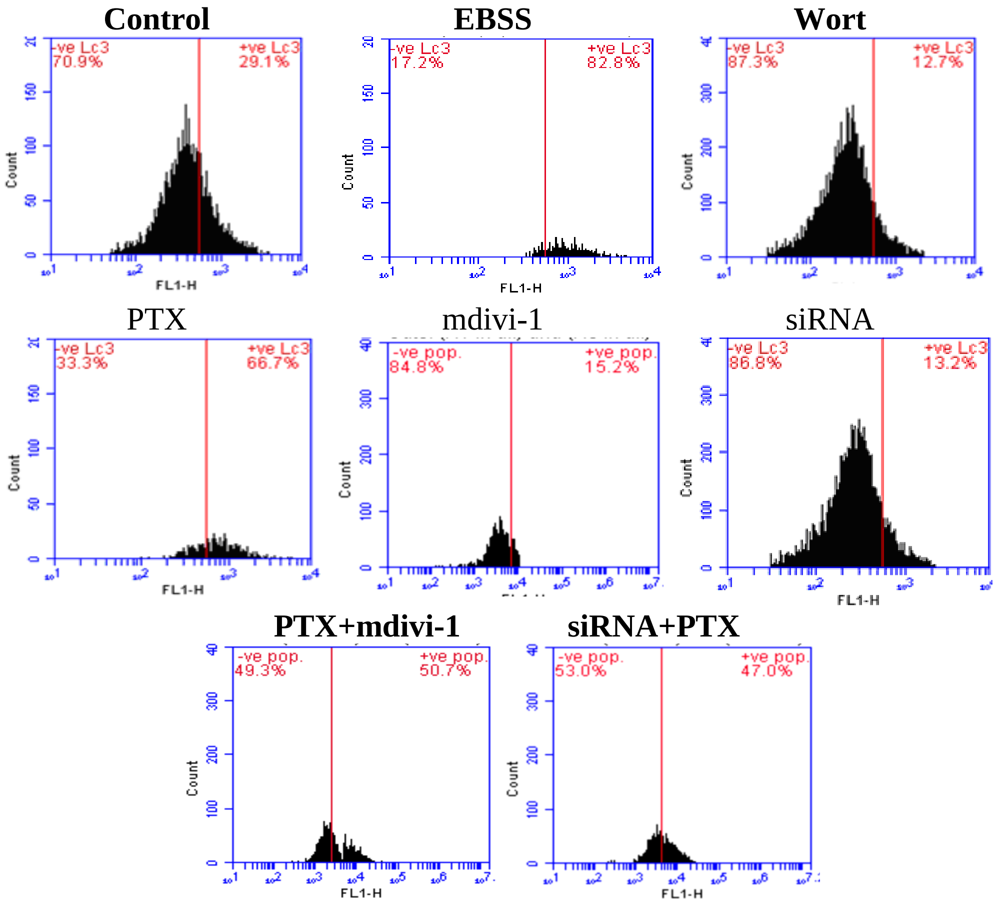

F

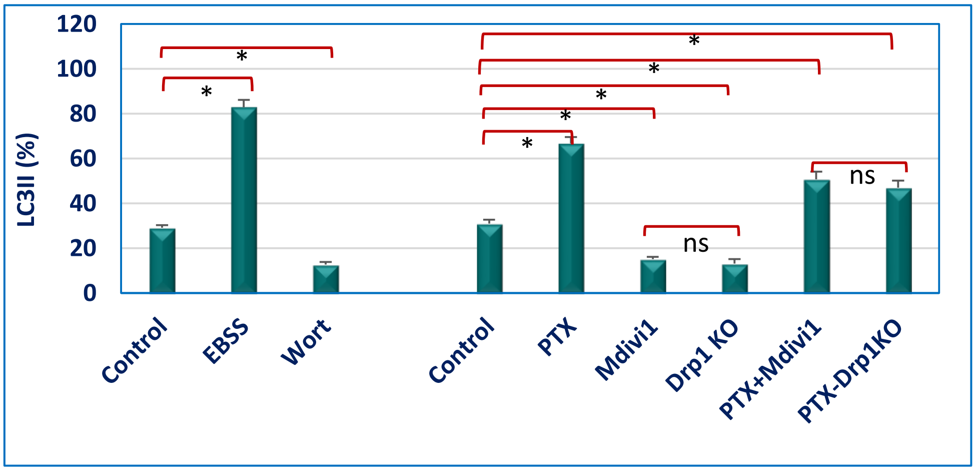

Figure 3A

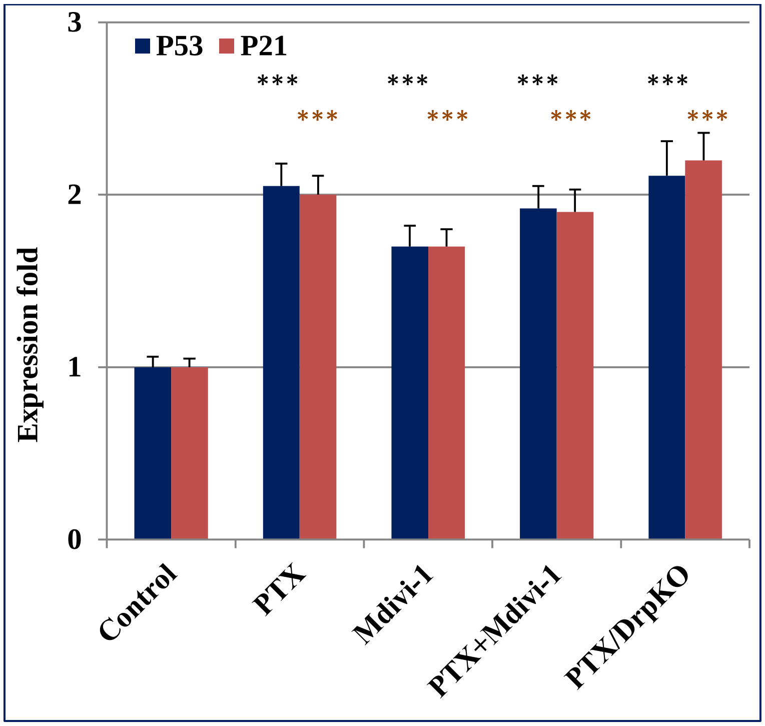

Comparison	Mean Difference	q		P value
Control vs PTX	-1.050	13.422	***	P<0.001
Control vs Mdivi-1	-0.7000	8.948	***	P<0.001
Control vs PTX+Mdivi-1	-0.9200	11.760	***	P<0.001
Control vs PTX/DrpKO	-1.110	14.189	***	P<0.001
PTX vs Mdivi-1	0.3500	4.474	ns	P>0.05
PTX vs PTX+Mdivi-1	0.1300	1.662	ns	P>0.05
PTX vs PTX/DrpKO	-0.06000	0.7670	ns	P>0.05
Mdivi-1 vs PTX+Mdivi-1	-0.2200	2.812	ns	P>0.05
Mdivi-1 vs PTX/DrpKO	-0.4100	5.241	*	P<0.05
PTX+Mdivi-1 vs PTX/DrpKO	-0.1900	2.429	ns	P>0.05

Figure 3B

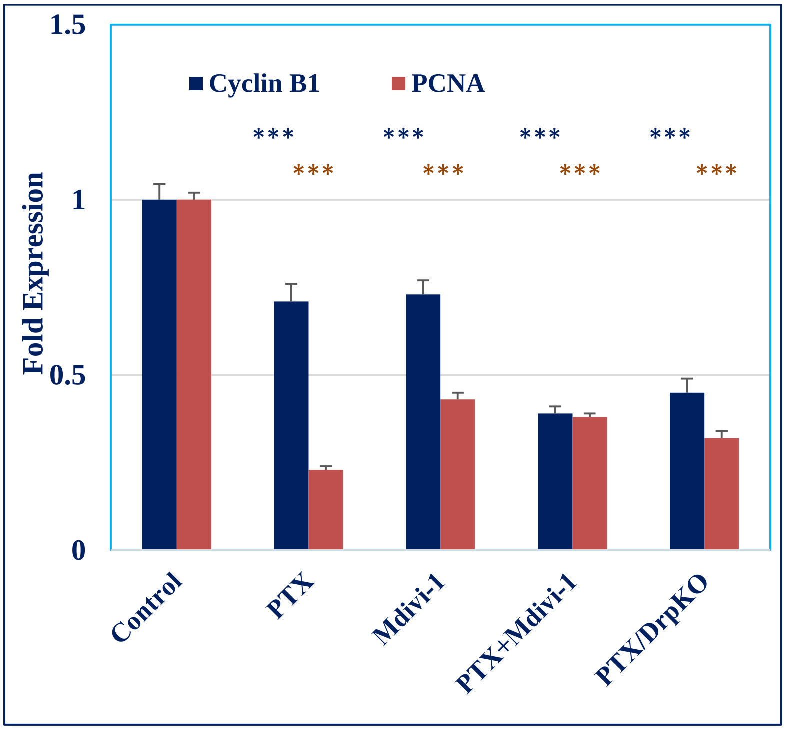

Comparison	Mean Difference	q		P value
Control-S vs Control-R	-0.2500	5.804	*	P<0.05
Control-S vs PTX	-0.2400	5.572	*	P<0.05
Control-S vs Mdivi-1	-0.2000	4.643	ns	P>0.05
Control-S vs PTX+Mdivi-1	8.004E-13	1.858E-11	ns	P>0.05
Control-S vs PTX/DrpKO	-0.04000	0.9286	ns	P>0.05
Control-R vs PTX	0.01000	0.2321	ns	P>0.05
Control-R vs Mdivi-1	0.05000	1.161	ns	P>0.05
Control-R vs PTX+Mdivi-1	0.2500	5.804	*	P<0.05
Control-R vs PTX/DrpKO	0.2100	4.875	*	P<0.05
PTX vs Mdivi-1	0.04000	0.9286	ns	P>0.05
PTX vs PTX+Mdivi-1	0.2400	5.572	*	P<0.05
PTX vs PTX/DrpKO	0.2000	4.643	ns	P>0.05
Mdivi-1 vs PTX+Mdivi-1	0.2000	4.643	ns	P>0.05
Mdivi-1 vs PTX/DrpKO	0.1600	3.714	ns	P>0.05

Figure 3C

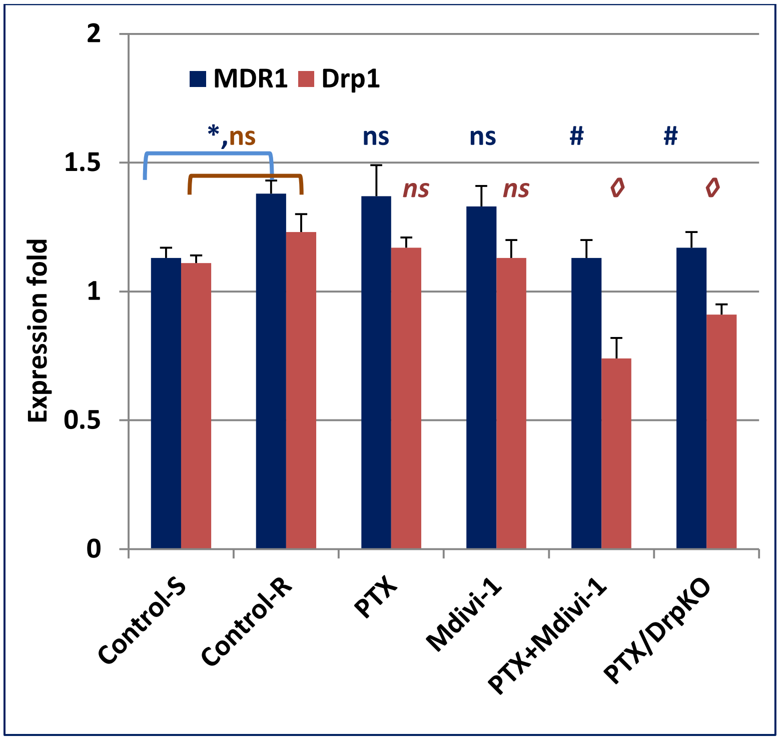

Comparison	Mean Difference	q		P value
Control-S vs Control-R	-0.1200	1.958	ns	P>0.05
Control-S vs PTX	-0.06000	0.9791	ns	P>0.05
Control-S vs Mdivi-1	-0.02000	0.3264	ns	P>0.05
Control-S vs PTX+Mdivi-1	0.3700	6.038	*	P<0.05
Control-S vs PTX/DrpKO	0.3000	4.895	*	P<0.05
Control-R vs PTX	0.06000	0.9791	ns	P>0.05
Control-R vs Mdivi-1	0.10000	1.632	ns	P>0.05
Control-R vs PTX+Mdivi-1	0.4900	7.996	**	P<0.01
Control-R vs PTX/DrpKO	0.4200	6.853	**	P<0.01
PTX vs Mdivi-1	0.04000	0.6527	ns	P>0.05
PTX vs PTX+Mdivi-1	0.4300	7.017	**	P<0.01
PTX vs PTX/DrpKO	0.3600	5.874	*	P<0.05
Mdivi-1 vs PTX+Mdivi-1	0.3900	6.364	**	P<0.01
Mdivi-1 vs PTX/DrpKO	0.3200	5.222	*	P<0.05

Figure 4

	Paclitaxel	Mdivi-1	Docking score/amino acids/bonding
**Mitochondrial ATP synthase**	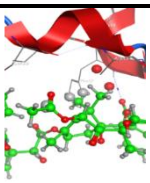<br>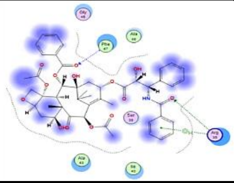	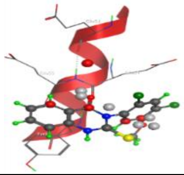<br>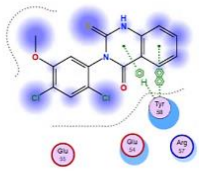	**PTX: -5.5581** (kcal/mol),<br>PHE-47 &ARG-35<br>(2 H-H bonds)<br>(pi-H) bond<br>**Mdivi1: -4.3050** (kcal/mol)<br>TYR 58 (pi-H) &<br>(pi_pi) bonds
**Superoxide demutase**	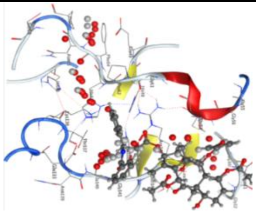<br>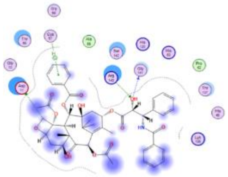	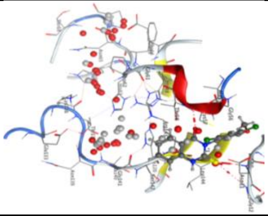<br>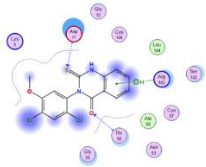	PTX: -7.3196<br>(kcal/mol)<br>GLY-141& ASP-11 &<br>ARG-143 & CYS 57<br>(3 H-H bonds)<br>(pi-H) bond bonds<br><br>Mdivi-1− 5.5982<br>(kcal/mol)<br>ASP-11 & THR-58<br>&ARG- 143<br>(2 H-H bonds)<br>(2pi-H) bond bonds
**Thioredoxin Reductase-1**	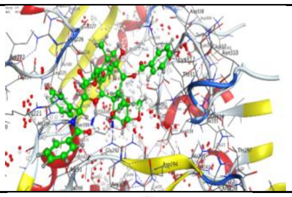<br>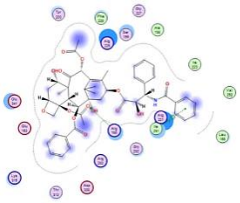	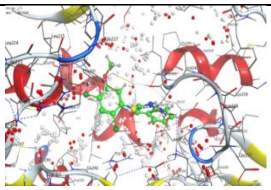<br>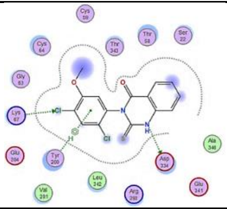	PTX: -8.76<br>(kcal/mol)<br>ARG-166 & ILE-291<br>(H-H bonds)<br>(pi-H) bonds<br>Mdivi-1: -7.0545<br>(kcal/mol)<br>ASP-334 & LYS-67<br>& TYR 200<br>(2 H-H bonds)<br>(pi-H) bonds
**Cisplatin (DDP)**		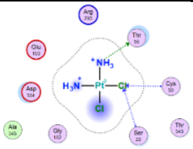	-4.4 kcal/mol<br>THR 58&<br>SER 22<br>CYS 59<br>(3H-H bonds)
**Carboplatin (CBP)**		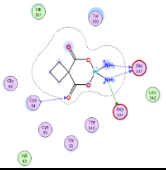	-5.13 kcal/mol<br>2 GLU-341 &<br>2 ASP-334&<br>CYS 64<br>(5 H-H bonds)
**Oxaliplatin (OHP)**		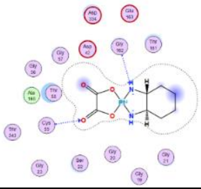	- 4.49 kcal/mol<br>GLY 162& CYS 59<br>(2 H-H bonds)

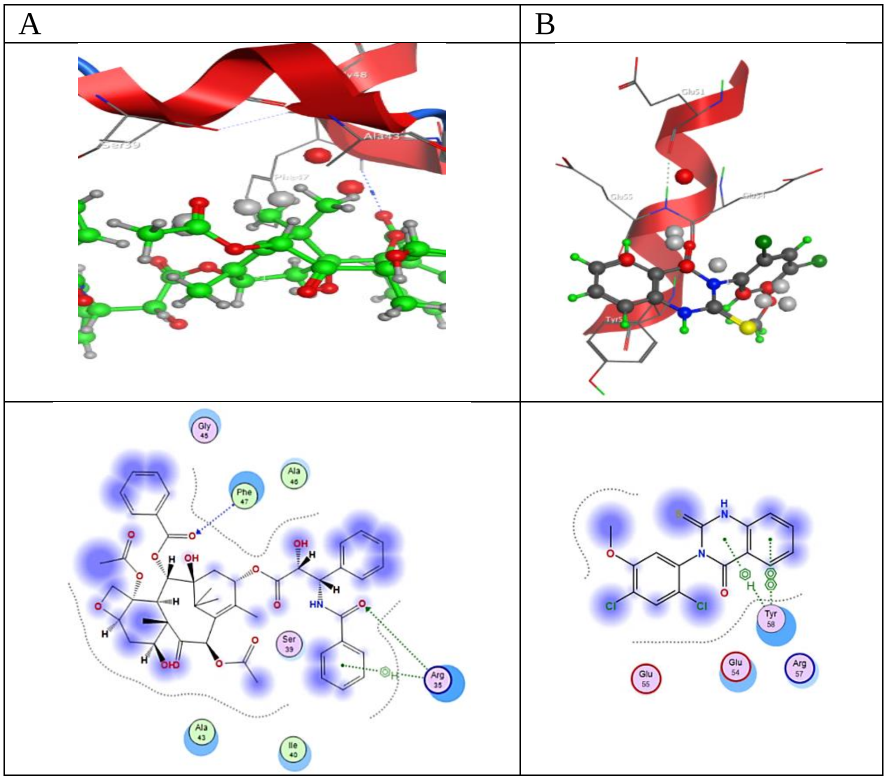

C

Compound	Docking score (kcal/mol)	Amino acids involved in binding
**Paclitaxel**	-5.5581 kcal/mol	PHE-47 &ARG-35<br>(2 H-H bonds)<br>(pi-H) bond
**Mdivi**	− 4.3050 kcal/mol	TYR 58 (pi-H) & (pi_pi) bonds

Compound ATPase	Docking score (kcal/mol)	Amino acids involved in binding
**Paclitaxel**	PTX=-5.5581 kcal/mol	PHE-47 &ARG-35<br>(2 H-H bonds)<br>(pi-H) bond
**Mdivi-1**	Mdivi1− 4.3050 kcal/mol	TYR 58 (pi-H) & (pi_pi) bonds

Compound SOD	Docking score (kcal/mol)	Amino acids involved in binding
**Paclitaxel**	-7.3196 kcal/mol	GLY-141& ASP-11 & ARG-143 &<br>CYS 57 (3 H-H bonds)<br>(pi-H) bond bonds
**Mdivi**	− 5.5982 kcal/mol	ASP-11 & THR-58 &ARG- 143<br>(2 H-H bonds)<br>(2pi-H) bond bonds

Compound TrxR	Docking score (kcal/mol)	Amino acids involved in binding
**Paclitaxel**	-8.7622 kcal/mol	ARG-166 & ILE-291<br>(H-H bonds)<br>(pi-H) bonds
**Mdivi-1**	− -7.0545 kcal/mol	ASP-334 & LYS-67 & TYR 200<br>(2 H-H bonds)<br>(pi-H) bonds

Fig 5 A (control)

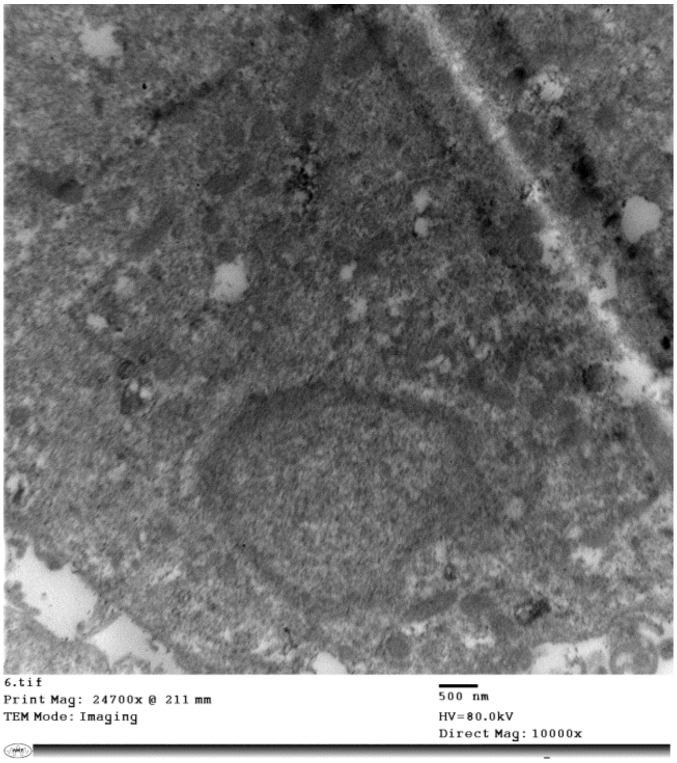

Fig 5 mivi-1

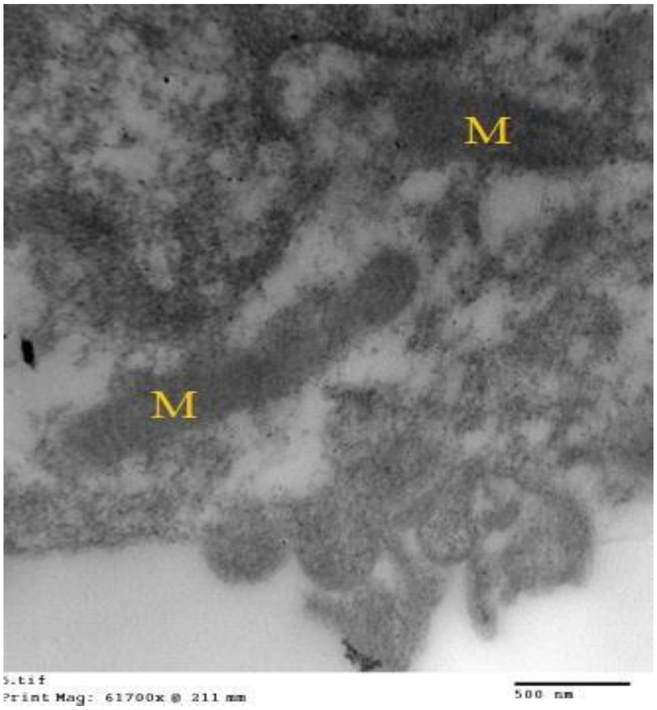

Fig 5 A PTX

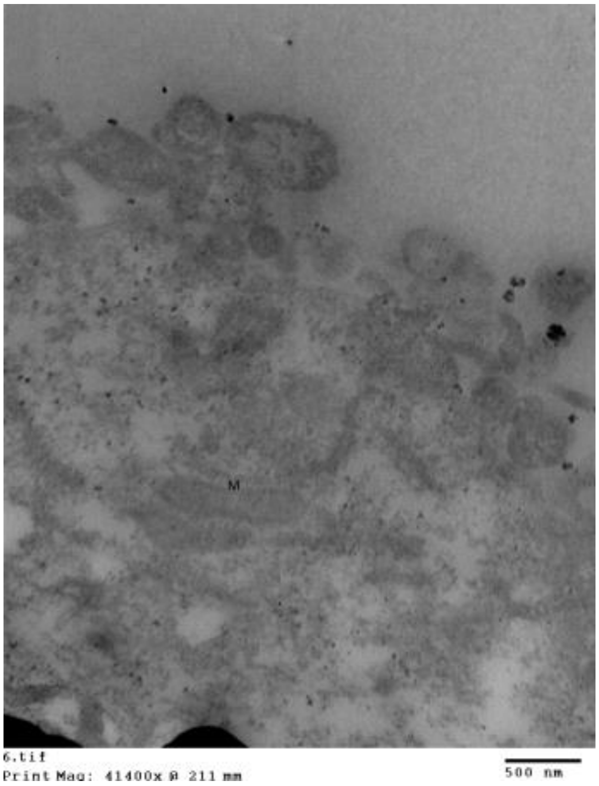

PTX+Mdivi-1

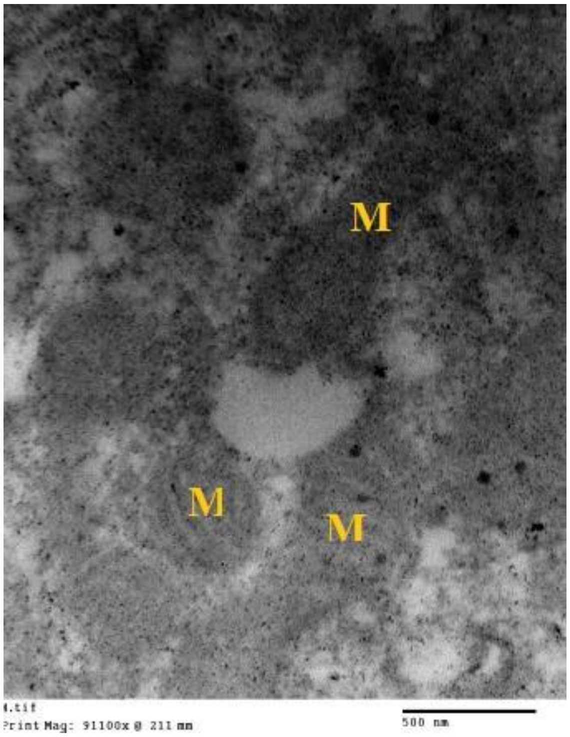

B

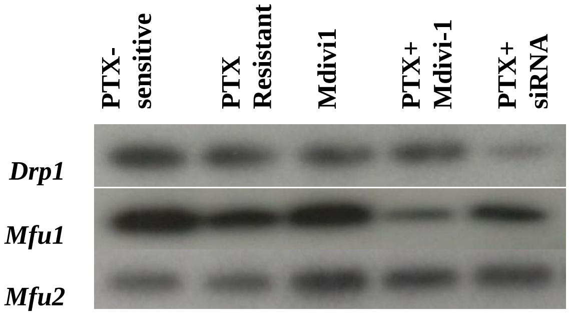

C

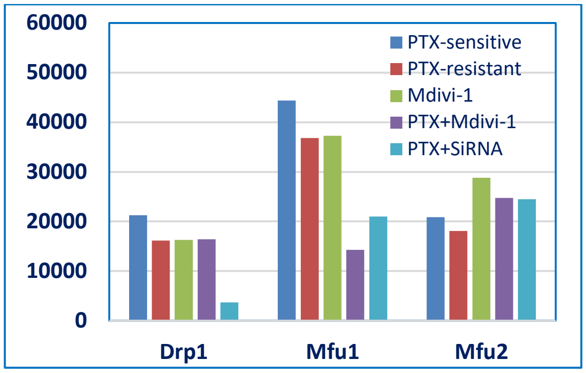

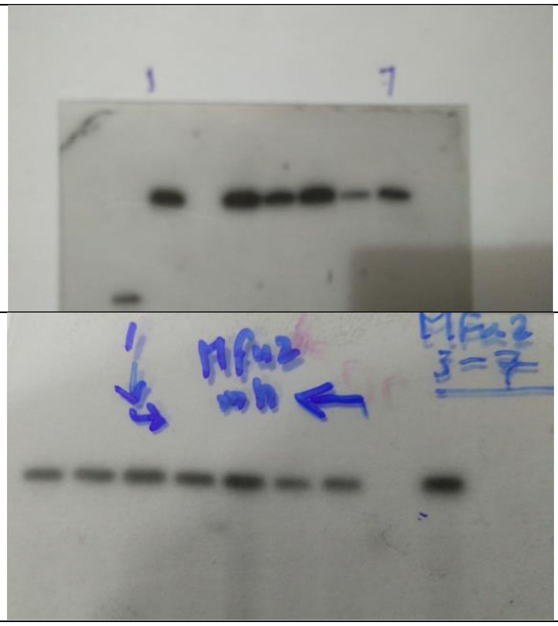

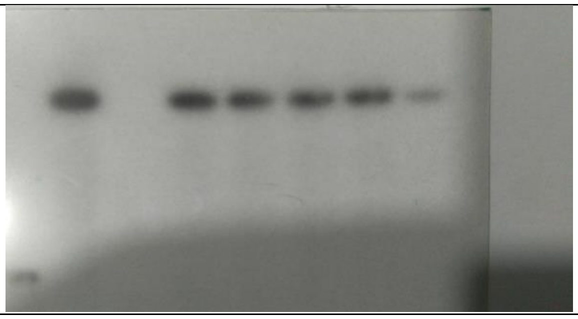

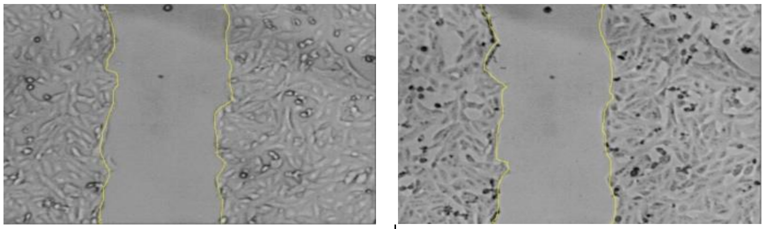

PTX+mdivi-1 siRNA+PTX

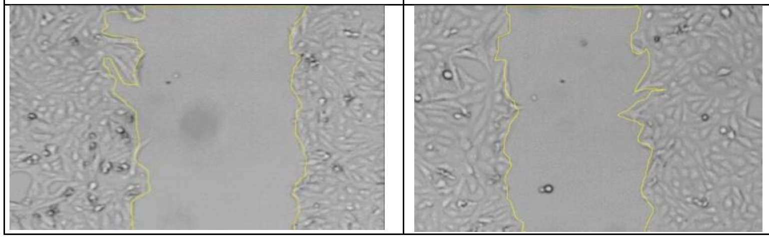

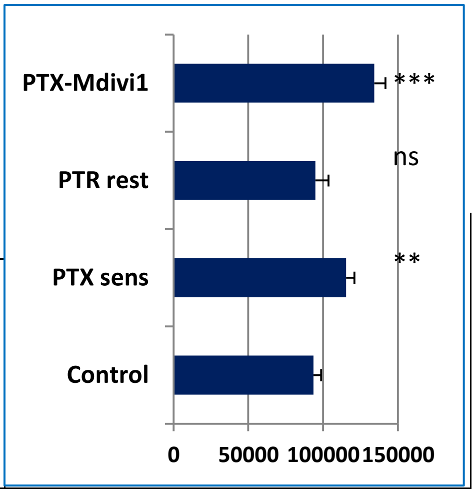

Figure 6A

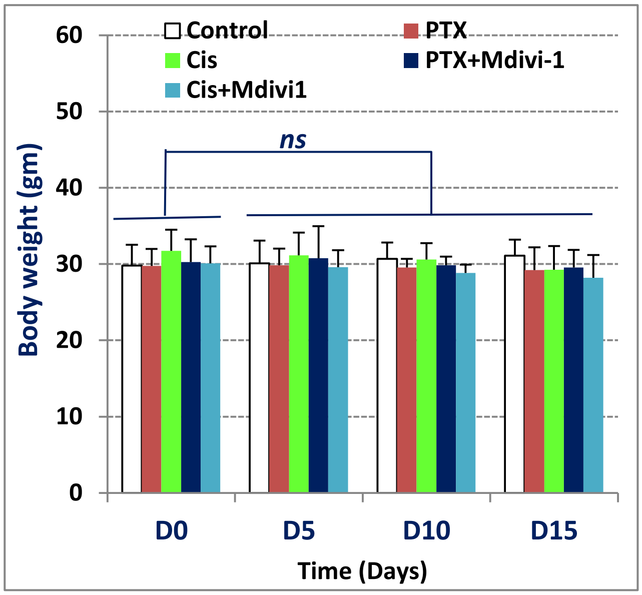

Figure 6B

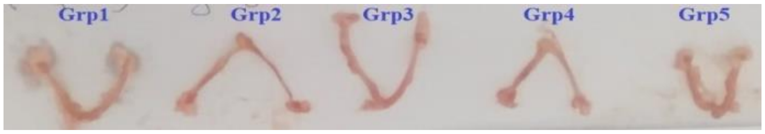

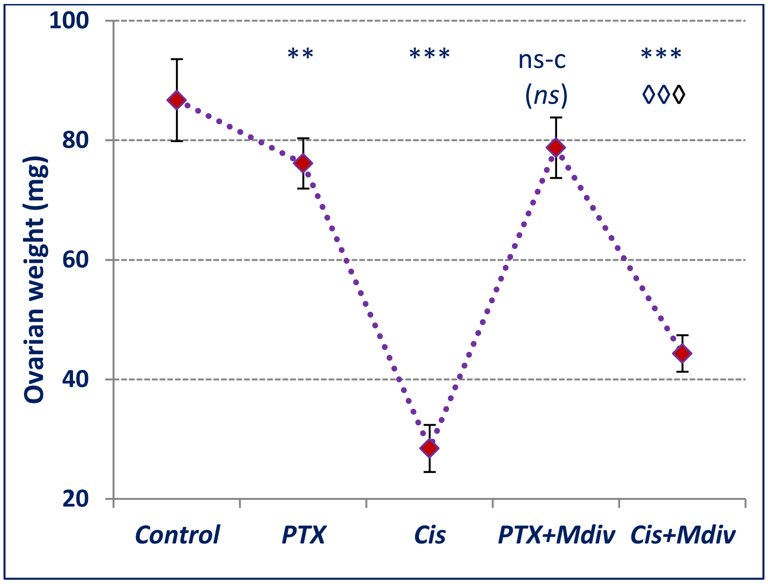

Figure 6C

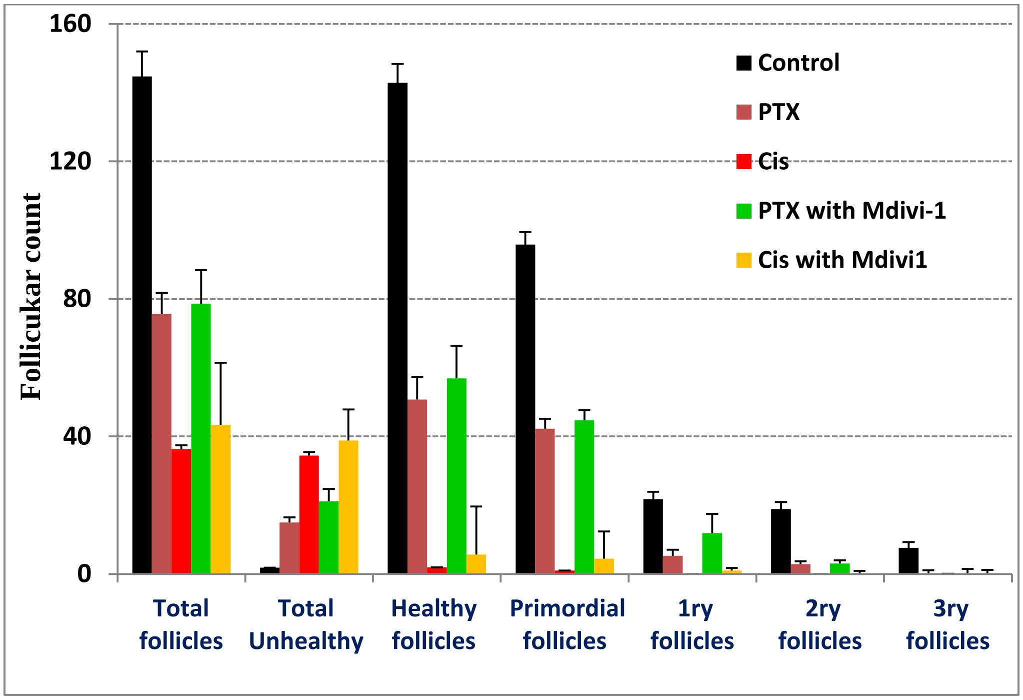

Figure 6D

Figure 6E

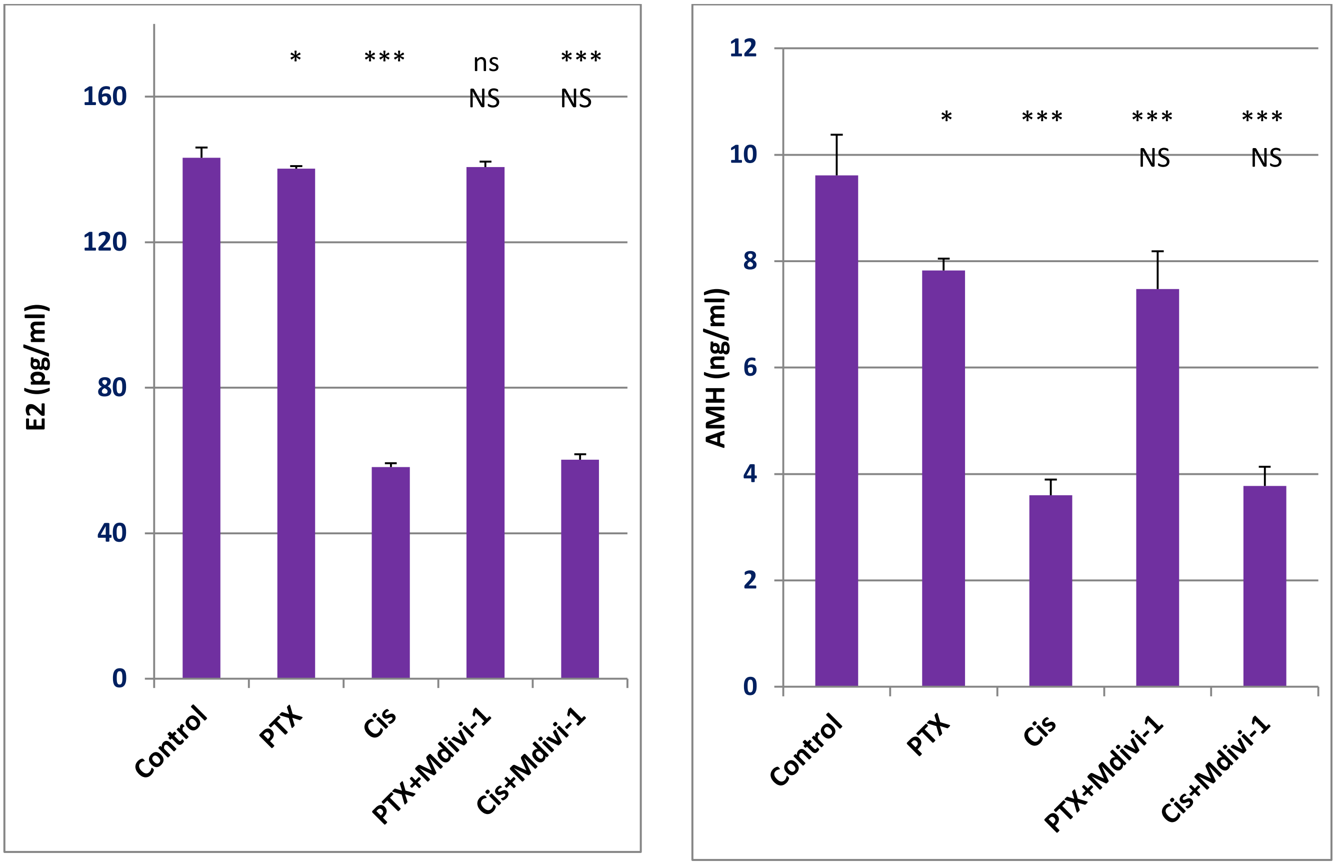
E2 (pg/ml)
0
40
80
120
160
*
***
ns
NS
***
NS
Control
PTX
Cis
PTX+Mdivi-1
Cis+Mdivi-1
AMH (ng/ml)
0
2
4
6
8
10
12
*
***
***
NS
***
NS
Control
PTX
Cis
PTX+Mdivi-1
Cis+Mdivi-1

Figure 7

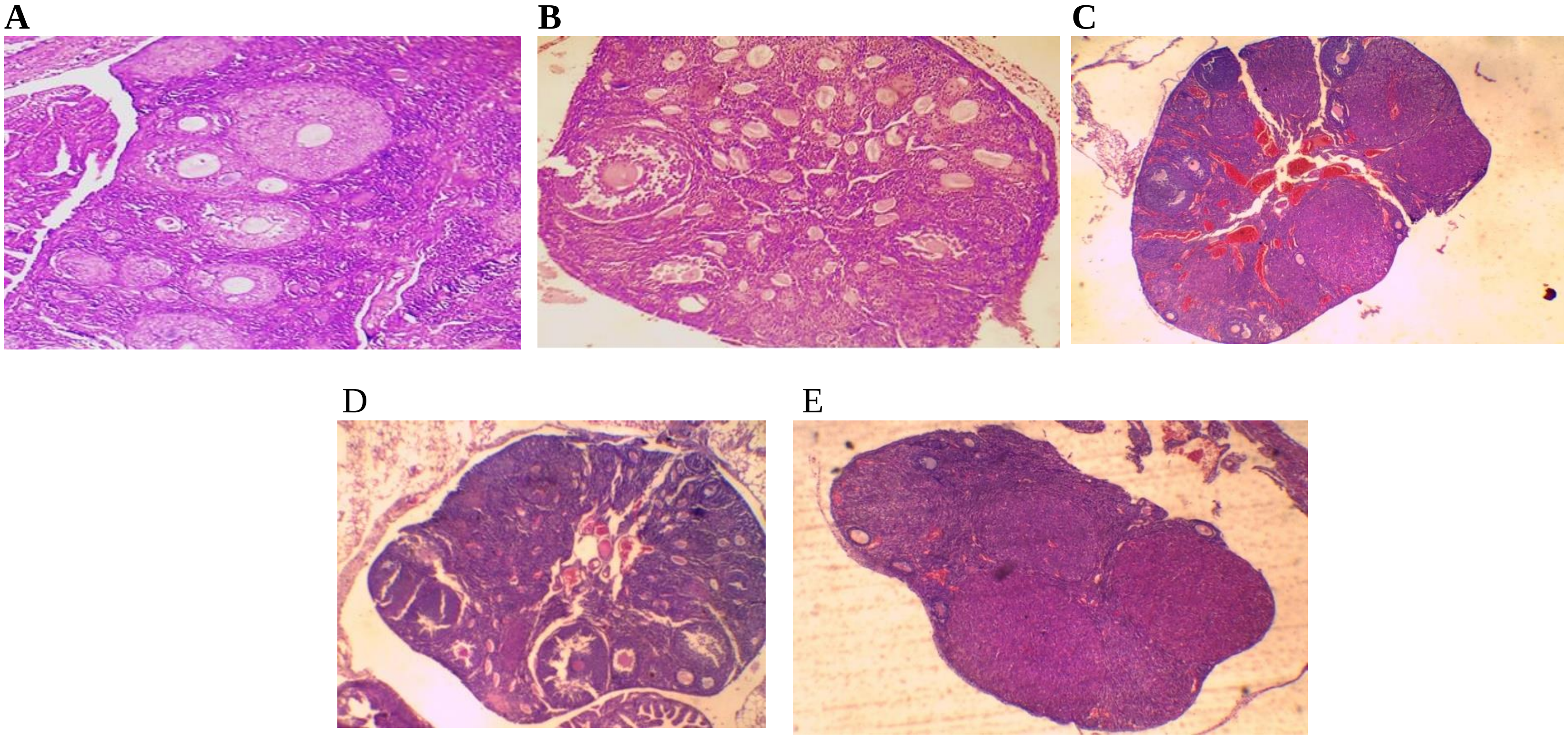

All panel resolutions

X400 DPI

Y 400 DP

Data not included in the manuscript.

Micromolar concentrations (50 µM), however, led to massive necrosis of the PTX-sensitive cells when they were exposed to PTX alone or combined with Drp1 inhibitor (Under Discussion section)

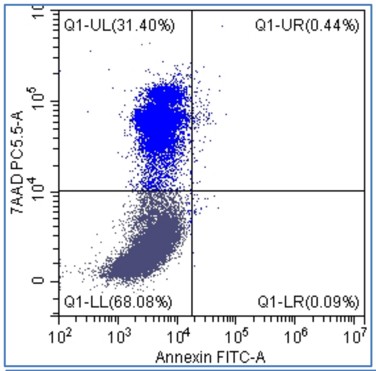

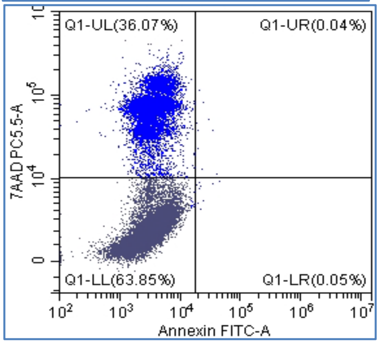

POF modeling

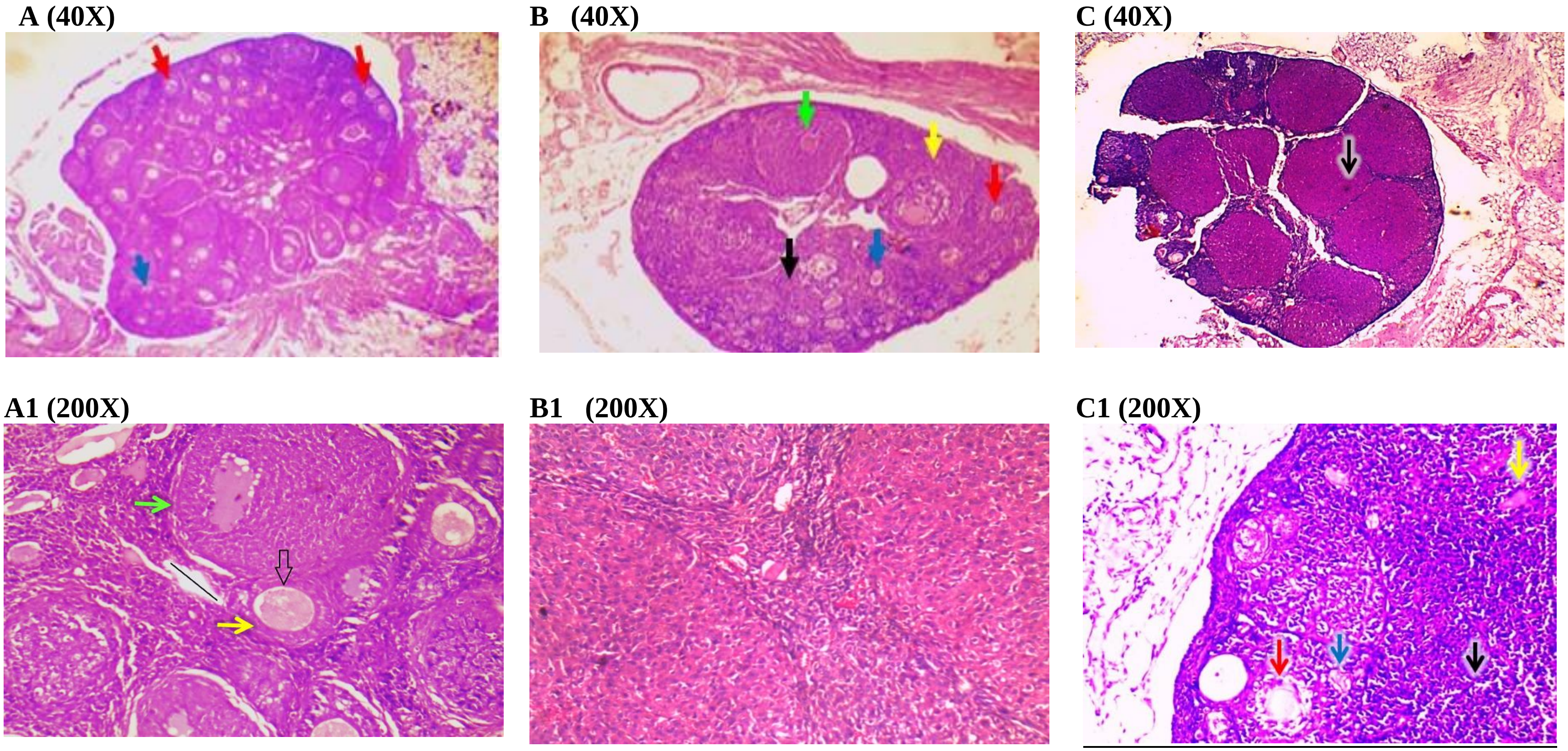

Fig. S1. Establishment of POF model. H&E staining of ovarian sections derived from healthy group (A, A1), POF mice generated by 2 mg/kg body weight cisplatin (B, B1), and POF mice intoxicated with 4 mg/kg body weight cisplatin (C, C1). Healthy animals demonstrate normal ovarian size and normal follicular count. Also, they showed healthy follicles in control group (A1). POF mice had a massive reduction in the number of follicles, development of few apoptosis in the granulomas cells in when POF was induced by 2 mg/kg cis (B1). Ovarian atrophy, severe reduction in the follicle member, abnormal oocyst and severe apoptosis in POF mice receiving 4 mg/kg (C1).
